# Zoonotic diseases of fish and their prevention and control

**DOI:** 10.1080/01652176.2022.2080298

**Published:** 2022-06-19

**Authors:** Mina Ziarati, Mohammad Jalil Zorriehzahra, Fatemeh Hassantabar, Zibandeh Mehrabi, Manish Dhawan, Khan Sharun, Talha Bin Emran, Kuldeep Dhama, Wanpen Chaicumpa, Shokoofeh Shamsi

**Affiliations:** a Department of Microbiology, Jahrom Branch, Islamic Azad University, Jahrom, I.R. Iran; b Department of Scientific Information and Communication, Iranian Fisheries Research Institute (IFSRI), Agricultural Research Education and Extension Organization (AREEO), Tehran, I.R. Iran; c Department of Fisheries, Faculty of Animal Science and Fisheries, Sari Agricultural Sciences and Natural Resources University, Sari, I.R. Iran; d Iran Fisheries Organization, Tehran, I.R. Iran; e Department of Microbiology, Punjab Agricultural University, Ludhiana, India; f The Trafford Group of Colleges, Manchester, UK; g Division of Surgery, ICAR-Indian Veterinary Research Institute, Bareilly, Uttar Pradesh, India; h Department of Pharmacy, BGC Trust University Bangladesh, Chittagong, Bangladesh; i Division of Pathology, ICAR-Indian Veterinary Research Institute, Bareilly, Uttar Pradesh, India; j Center of Research Excellence on Therapeutic Proteins and Antibody Engineering, Department of Parasitology, Faculty of Medicine Siriraj Hospital, Mahidol University, Bangkok, Thailand; k School of Animal and Veterinary Sciences, Charles Sturt University, Wagga Wagga, NSW, Australia

**Keywords:** Fish, aquaculture, zoonosis, prevention, control

## Abstract

Fish and aquatic-derived zoonotic diseases have caused considerable problems in the aquaculture industry and fishery worldwide. In particular, zoonotic diseases can pose widespread threats to humans. With the world’s growing population and potential global trade of aquaculture and fish, the risk of environmental contamination and development of fish and aquatic-derived zoonoses in humans are increasing. The important causes of zoonoses include bacteria, parasites, viruses, and fungi. The zoonotic bacterial agents are divided into two main groups: Gram-positive (*Mycobacteriaceae*, *Streptococcaceae*, *Erysipelothricaceae* families) and Gram-negative (*Aeromonadaceae*, *Vibrionaceae*, *Pseudomondaceae*, *Enterobacteriaceae*, and *Hafniaceae* families). The premier parasitic agents include cestodes (tapeworm; e.g. *Diphyllobothrium* spp.), trematodes (fluke; e.g. *Opisthorchis* spp.), and nematodes (round worm; e.g. *Anisakis* spp.). In addition, protozoan organisms such as *Cryptosporidium* spp. are also considered fish-derived zoonotic pathogens. Two groups of fish-associated fungi causing basidiobolomycosis and sporotrichosis also pose a zoonotic risk for humans. The majority of the fish-derived zoonotic diseases are transmitted to humans mainly via the consumption of improperly cooked or raw fish or fish products. Therefore, the incidence of zoonotic diseases can be reduced by properly processing fish and fish products, e.g. by thermal (heat/freezing) treatment. The prevalence of zoonotic agents in fishes varies seasonally and should be regularly monitored to evaluate the prevalence of pathogens in both wild and cultured fish populations. This review focuses on the fish zoonotic agents/diseases and their control and prevention.

## 1. Introduction

A zoonosis (zoonotic disease; zoonoses for pleural) is an infectious disease that is transmitted between animal species to humans (Han et al. 2016). Several causative agents of infectious diseases, including bacteria, viruses, parasites, and fungi, can be transmitted from animals to people through different routes, including penetration through wounded or abrasive skin, ingestion, animal bites, vectors (i.e. insects), and animal-to-human contact (i.e. inhalation of respiratory particles or skin/mucous membrane contact) (Gauthier 2015; Rahman et al. 2020). The pathogens that usually exist in animals can infect humans either directly or via a vector (Wolfe et al. 2007). Within aquatics, the general perception is that there are few zoonotic diseases considered as important (Shamsi 2019). For those that are detected, the number of cases per year is small compared to other zoonotic diseases in animals or humans, such as campylobacteriosis or salmonellosis. While this might be correct, there is a possibility that this is an underestimate due to poor awareness and lack of monitoring and surveillance. However, for those that are diagnosed, the consequences can be severe, including death (Zorriehzahra and Talebi 2021).

In a similar study, it was confirmed that about 260,000 people get sick from contaminated fish in the USA per year. Fish meat is often the most commonly implicated food category in the mentioned outbreaks. The Foodborne Disease Outbreak Surveillance System (FDOSS) of CDC is involved in collecting data on foodborne disease outbreaks. Also, about 857 outbreaks were associated with fish, resulting in 4815 illnesses, 359 hospitalizations, and deaths (Barrett et al. 2017). These hazardous fish zoonotic outbreaks reported over the years (Table 1) indicate the importance of monitoring fish-derived zoonotic diseases.

**Table 1.** Some important fish zoonotic outbreaks in the recent decade.

No.	Outbreak name	Main pathogen	Host	Reference
1	Two listeriosis outbreaks caused by smoked fish consumption	*Listeria monocytogenes*	Smoked fish	Lassen et al. 2016
2	2015 Epidemic of severe *Streptococcus agalactiae* sequence type 283 infections in Singapore associated with the consumption of raw freshwater fish	*Streptococcus agalactiae*	Asian bighead carp (*Hypophthalmichthys nobilis*) and snakehead fish (*Channa* species)	Kalimuddin et al. 2017
3	Outbreak of tularemia associated with crayfish fishing	*Francisella tularensis*	Crayfish	Anda et al. 2001
4	Two outbreaks of botulism infection after eating fish in Norway and Germany	*Clostridium botulinum*	Rakfish	Eriksen et al. 2004
5	Large outbreak of *Salmonella* Thompson related to smoked salmon in the Netherlands	*Salmonella enterica*	Smoked salmon	Friesema et al. 2014
6	Acute outbreak of gnathostomiasis in a fishing community in Sinaloa, Mexico	*Gnathostoma binucleatum*	Spotted sleeper perch (*Eleotrispicta*)	Camacho et al. 2003

Many diseases found in aquatic animals can be classified as emerging diseases, defined by the World Health Organization (WHO) as, ‘an emerging disease has appeared in a population for the first time, or that may have existed previously but is rapidly increasing in incidence or geographic range’. One attribute of emerging diseases is that information on the zoonotic potential is limited. Yet, where a possibility exists, it is essential to ensure that information is disseminated to other professionals and the public effectively and quickly. This can be done by a qualitative risk assessment. Questions that need to be answered in carrying out the assessment include the etiology, geographical distribution, prevalence, incidence, eco-epidemiology, clinical symptoms, availability of diagnostic tests, assessment of zoonotic potential, the potential sources of human exposure and, detection of zoonotic disease potential (Zorriehzahra et al. 2014; Farzadnia and Naeemipour 2020).

In recent years, with the increase in the world population and the consumption of seafood, demand for seafood has increased. Since seafoods are one of the sources of protein for people, the fisheries and aquaculture industry have also shown sustainable growth worldwide; however, they are not risk-free (Shamsi 2019; Tran et al. 2019). In addition to food poisoning from seafood, there is also the transmission of aquatic pathogens to humans. Several important factors in fish and the water around them have shown the potential for disease transmission to humans (Environmental Health and Safety (EHS)/Occupational Health 2016; Raissy 2017). The immune system plays a vital role in determining the severity of aquatic zoonotic diseases. Nevertheless, there are specifically two main ways of human diseases. First, eating raw or undercooked fish and swallowing water or other matters contaminated with infected fish feces/mucus. Second, contact with the infectious agent through open wounds or skin scratch/abrasion. According to Raissy (2017), 46% of fish-derived zoonotic diseases are transmitted orally while 15% have more than one transmission route. Transmission via consumption of water with infected organisms and skin contact during the handling of fish are 24 and 19%, respectively (Raissy 2017).

Although contamination of humans with fish pathogens is uncommon, it should be considered as a serious risk to human health (Aggarwal and Ramachandran 2020). In the meantime, zoonotic diseases have been identified as a source of human emerging infectious diseases (Jones et al. 2008). Emergence of zoonotic agents is a serious threat to global health and causes great damage worldwide (World Health Organization (WHO) 2021). The COVID-19 pandemic has once again emphasized the significance of the human and animal interaction in the spread of zoonotic illnesses, particularly wildlife and livestock species that serve as potential hosts and viral reservoirs. Therefore, it is essential to identify the particular factors and mechanisms that lead to disease emergence to deal with emerging infectious diseases. In the face of globalization, habitat loss, climate changes, and interconnections among wildlife and livestock systems can contribute to the spread of zoonotic diseases (Meurens et al. 2021).

**Figure 1.**
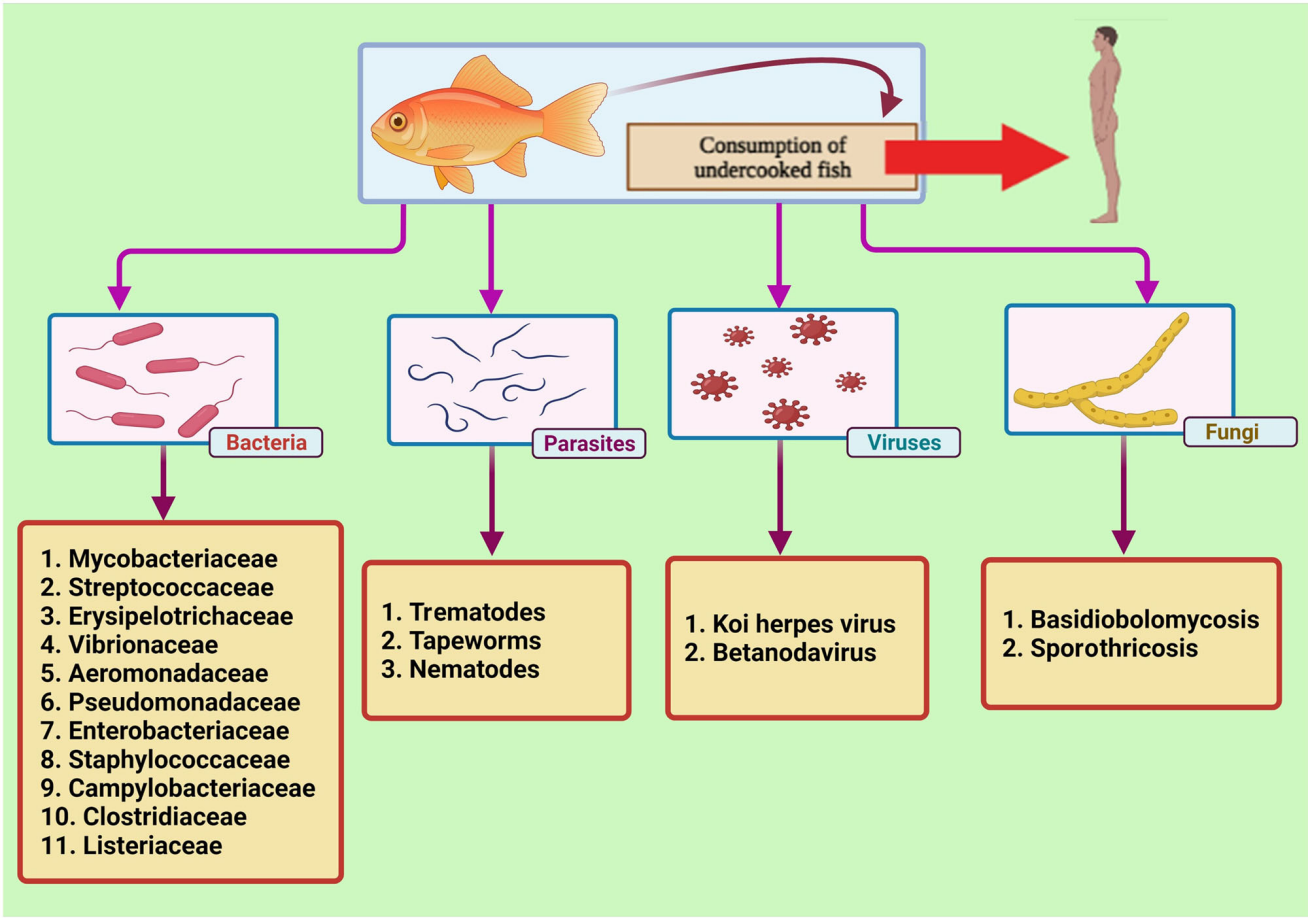
A broad classification of fish zoonotic agents including bacteria, parasites, viruses, and fungi. The figure was created with BioRender.com.

Various factors such as the type of microorganisms (bacteria, viruses, parasites, fungi), the host status (existence of open wounds on body, penetrated by spines, immunocompromised), and the environmental factors (contaminated water) are involved in the transmission of fish pathogens to humans (Haenen et al. 2013). Among the fish-associated pathogens, the most important infectious agents are bacteria, parasites, and viruses (Shamsi 2019; Meurens et al. 2021). In addition, protozoan organisms such as *Cryptosporidium* spp. are also considered as a fish-derived zoonotic risk for humans. Several *Cryptosporidium* species have been identified in freshwater, cultured, marine, and ornamental fishes worldwide (Golomazou et al. 2021). The rapid increase of fisheries and aquaculture globally and, on the other hand, the existence of transmissible agents from fish to humans has caused the present review to focus on the bacterial, virus, parasitic and fungal zoonotic diseases, the risk to human and ultimately their control and prevention. Figure 1 shows the types of pathogens transmitted from fish to humans.

## 2. Bacterial zoonotic agents

The main zoonotic agents of fish are bacteria, which are divided into two main groups. The majority are Gram-negative bacteria and few Gram-positive bacteria (Smith 2011; Gauthier 2015). Apparently healthy fishes may also harbor bacterial pathogens, especially in their kidneys and intestines (Meron et al. 2020). Infection due to *Vibrio* and *Mycobacterium* species is considered the major cause of economic loss and occasionally represented as the limiting factor in fish production (Regev et al. 2020). The prevalence of bacterial zoonotic agents in fishes varies annually and should be continuously monitored to evaluate the prevalence of pathogens in both wild and cultured fish populations (Meron et al. 2020; Regev et al. 2020). In addition, ornamental fishes can also act as an important source of bacterial zoonotic agents that also exhibit high levels of antimicrobial resistance (Weir et al. 2012).

### 2.1. Mycobacteriaceae

*Mycobacterium* spp. are Gram-positive, acid-fast, aerobic, non-motile pleomorphic bacilli belong to the *Mycobacteriaceae* family, which includes many pathogenic bacteria related to humans, mammals, reptiles, and fish (Delghandi et al. 2020a, 2020b). Mycotuberculosis is a common disease of marine, freshwater, and brackish water fish and is considered a major cause of mortality of farmed and free-living fish (Hashish et al. 2018). Non-tuberculosis *Mycobacterium* (NTM) infections of fish have been identified in more than 150 fish species worldwide, and its zoonotic nature is a public health concern (Gcebe et al. 2018). Most species of fish are susceptible to *Mycobacterium*, and it can transmit the bacteria horizontally and vertically. Occurrence of *Mycobacterium* spp. has also been reported in ornamental fish (Puk and Guz 2020). The clinical signs of infected fish are different due to the numerous species of the bacteria and the wide range of host species (Delghandi et al. 2020a). The specific signs of fish infected with *Mycobacterium* species are lethargy, pigmentation, abdominal distention, exophthalmia, skin lesions, and death (Smith 2011). However, due to the spread of infection through the circulatory and lymphatic system, the infection is found in some fish organs like eyes, gills, liver, kidneys, spleen (Chinabut 1999). The manifestations of infected fish also can include enlarged liver, kidney, spleen, and nodules in internal organs (Delghandi et al. 2020a). Infected and asymptomatic fish are carriers or spreaders of the bacteria in the long term (Boylan 2011), and human diseases often occur in contact with infected aquatics and water (Bhambri et al. 2009). About 120 species of *Mycobacterium* are known; the most important causes of fish zoonoses are *M. avescencs*, *M. chelonae*, *M. fortuitum*, *M. gordonae*, *M. marinum*, *M. ulcerans*, *M. septicum*, *M. peregrinum* and *M. avium*. Their infections can lead to acute and chronic illness (Smith 2011; Delghandi et al. 2020a). The disease in humans caused by these mycobacteria usually leads to granulomatous lesions on the skin, severe necrotic lesions, and deep-tissue infections such as tendons and bones. Still, systemic respiratory and extra-respiratory diseases are rare but can occur in immunocompromised patients. Occasionally, arthritis, osteomyelitis, and bronchitis occur (Delghandi et al. 2020a). Mycobacteriosis in immunocompromised patients can progress to systemic infection and death (Boylan 2011). *Mycobacterium* virulence factors include type VII secretory system, ESX genes, accessibility to the cytosol and activation of host actin polymerization for motility and cell-to-cell migration (Hashish et al. 2018). Table 2 shows the most important *Mycobacterium* spp. in the freshwater, marine and ornamental fish. Among the various species of *Mycobacterium*, four species are the most common and play a key role in the occurrence of outbreak. These four species consist of *M. marinum*, that has most importance role and then other fish mycobacterial pathogens, *M. fortuitum*, *M. gordonae*, and *M. chelonae*.

**Table 2.** The most common *Mycobacterium* spp. isolated from fish in the different environments.

Aquatic Host	*Mycobacterium* species
**Freshwater Fish**	*M. abscessus*, *M. avium*, *M. chelonae*, *M. flavescens*, *M. fortuitum*, *M. haemophilum*, *M. lentiflavum*, *M. marinum*, *M. nonchromogenicum*, *M. peregrinum*, *M. salmoniphilum*, *M. septicum,* *M. shottsii*, *M. smegmatis*, *M. stephanolepidis*
**Marine Fish**	*M. chesapeaki*, , *M. chelonae*, *M. gordonae*, *M. holsaticum*, *M. marinum*, *M. montefiorence*, *M. neoaurum*, *M. pseudoshottsii*, *M. syngnathidarum*
**Ornamental Fish**	*M. avium*, *M. abscessus,M. chelonae, M. fortuitum,* *M. gordonae*, *M. marinum*, *M. mucogenicum*, *M. neoaurum*, *M. peregrinum*, *M. salmoniphilum, M. saopaulense, M. senegalense, M. septicum, M. shimoidei, M. szulgai, M. triviale*

Piscine mycobacteriosis was detected in the ornamental fishes used for trading in Trinidad and Tobago (Phillips Savage et al. 2022). The presence of *Mycobacterium* spp. in the freshwater ornamental fishes sold in pet stores poses a great risk to the individuals handling it. One health approach is necessary to detect the presence of such zoonotic pathogens and to prevent their onward transmission to human beings.

### 2.2. Streptococcaceae

Another Gram-positive bacterial family and zoonotic agent is *Streptococcaceae*. This bacterial family causes systemic streptococcosis, which has become a threat to fish worldwide, and inflicts economic damages, and public health concerns (Iregui et al. 2016). The bacteria in the family are considered emerging zoonotic agents and human pathogens during contact with the fish (Ziarati et al. 2018). There have been reports of meningoencephalitis and death in farmed fish species (Novotny et al. 2004). Furthermore, the bacteria have created high morbidity and mortality among fish of fresh- and salt-water. Both horizontal and vertical transmission have been reported.

Routes of transmission to human include direct contact with a disease or dead fish and indirect contact with contaminated water. The principal bacteria that cause fish streptococcosis include *S. agalactiae*, *S. difficile*, *S. difficilis*, *S. dysgalactiae*, *S. iniae*, and *S. shiloi* (Pradeep et al. 2016). Moreover, group B *Streptococcus* (GBS) ST283 strains have been detected in freshwater and marine fish, humans, and frogs (Barkham et al. 2019; Zadoks et al. 2020). Clinical signs of the disease depend on the fish species. Still, the most frequent manifestations are exophthalmia, abdominal distention, loss of orientation, erratic swimming, anorexia, eye opacity, darkening and hemorrhagic skin, and eventually death (Karsidani et al. 2010; Boylan 2011; Leal et al. 2019). *Streptococcus* is considered a neurotropic agent for fish based on the clinical symptoms and unbalanced swimming behavior. The bacteria are found in the liver, gills, kidneys, spleen, and tissues required for fish defense against pathogens. Research on *Streptococcus* pathogenicity has shown that the gastrointestinal tract was the main portal of entry for *S. agalactiae* in tilapia, and the bacterium could cross mucosa and intestinal layers (Iregui et al. 2016). *Streptococcus* virulence factors include surface proteins, capsular polysaccharides, and secreted products. Bacterial surface proteins bind to human fibrinogen to inhibit phagocytic activity. They can also bind to immunoglobulins. The bacterial peptidase C5 and protease degrade complement component C5A and interleukin-8 chemokine, respectively, thereby destroying chemotactic signals and hence disrupts phagocyte recruitment. The bacterial streptolysin destroys lymphocytes, erythrocytes, and neutrophils. The synthesis of polysaccharides around the cell and extracellular exopolysaccharides contribute to greater adhesion (and resistance to toxic substances). The bacterial α-anolase destroys fibrin clots and facilitates bacterial spreads (Baiano and Barnes 2009). In humans, handling of infected live and killed fish can result in development of cellulite, endocarditis, meningitis, severe systemic infections, suppurating ulcers, septicemia, arthritis, lymphadenitis (Haenen et al. 2013), and rarely death (Smith 2011). *Streptococcus iniae* (first isolated from Amazon freshwater dolphin in 1970s) is marine pathogenic bacteria worldwide and is considered as one of the major threats to marine aquaculture due to its high prevalence (an overall prevalence of about 10% in wild marine fish and crustaceans sampled from the Mediterranean Sea) (Berzak et al. 2019).

### 2.3. Erysipelotrichaceae

*Erysipelothrix* is a Gram-positive bacterium associated with fish zoonoses. It has relation with sea mammals and causes skin diseases or acute sepsis (Boylan 2011). The most important member of this family is *E. rhusiopathiae* (formerly known as *E. insidiosa)*, which causes disease in animals and humans and trends for targeting the skin, connective tissues, and vascular walls. Clinical manifestations include necrotizing dermatitis, myositis, and cellulitis. Before 2014, *E. rhusiopathiae* was thought to be a common bacterium of fish, but *Erysipelothrix*-associated mortality has been reported in various countries. Recently, an emerging species of ornamental fish, *E. piscisicarius* has been reported in fish (Pomaranski et al. 2020). This organism is a soil saprophyte that grows well in fish and can easily cause erysipeloidin fish sellers/handlers in hot seasons (Novotny et al. 2004). Human gets the bacterial infection when fish mucus containing the bacteria is transmitted to humans by touching fresh or dead fish (Boylan 2011). It should be noted that *E. rhusiopathiae* does not cause any disease in fish. Still, due to its long-term survival on the fish outer mucus, it can transmit to humans and cause erysipeloid disease (Nielsen et al. 2018). Therefore, human infections caused by *Erysipelothrix* are related to contact with infected animals and their products or waste (Ahmadi Balootaki et al. 2017). *E. rhusiopathiae* causes septicemia, skin infection (mainly hands), and endocarditis. Fishers and veterinarians are among people at high risk to *Erysipelothrix* infections. In 2017, the first human endocarditis disease was reported due to this bacterium, which was associated with work off the coast of Norway (Nielsen et al. 2018).

### 2.4. Vibrionaceae

*Vibrio* species, as Gram-negative bacteria that cause vibriosis in animals and humans, posing a risk of zoonotic in aquaculture professionals and consumers of aquatic products (Austin 2010). On the other hand, because vibriosis is a potentially dangerous disease in fish, the high use of antibiotics in cultured systems has increased antibiotic resistance in the bacteria (Helmi et al. 2020). *Vibrio* species are also abundant in brackish- and fresh-water, and human infections by them occur through skin lesions and ingestion of contaminated fish. The following species infect humans: *V. cholerae*, *V. alginolyticus*, *V. vulnificus*, *V. damselae*, *V. hollisae*, *V. metschnikovi*, and *V. parahaemolyticus* (Boylan 2011). Some important species found in infected fish include *V. alginolyticus*, *V. anguillarum*, *V. campbellii*, *V. harvey*, *V. vulnificus*, and *V. parahaemolyticus* (Huzmi et al. 2019). The premier species of *Vibrio* in marine fish are *V. vulnificus* and *V. parahaemolyticus*, and the most common human infections are caused by *V. cholerae*, *V. vulnificus* and *V. parahaemolyticus*. Clinical signs of *Vibrio*-infected fish are often nonspecific and include lethargy, skin lesions, exophthalmia, and death (Smith 2011). In addition, other symptoms, such as swollen spleen, abdominal dropsy, intestinal inflammation, epidermal bleeding, scale shedding, pop-eye, and tail rot, have been reported (Huzmi et al. 2019). Vibriosis consists of three main stages (entering through the skin, fins, gills, and anus; destruction of tissue and host cells; and exit) that can affect the host and lead to mortality. Some *Vibrio* virulence agents include siderophores, extracellular products (ECPs), hydrolytic enzymes and toxins. Resistance to vibriosis depends on the interaction of pathogen, host, and environment, but there are reports of about 100% fish mortality with some *Vibrio* species (Jun and Woo 2003). Transmission of *Vibrio* species from fish to humans can cause diseases such as lesions, septicemia, erythema, and tissue necrosis (Hernández-Cabanyero and Amaro 2020). The increasing preference of consumers toward ready-to-eat seafood items such as raw fish flesh slices can cause seafood-derived illness due to *V. parahaemolyticus* (You et al. 2021). *V. vulnificus* is an important zoonotic pathogen of public health concern. It is reported to cause primary septicemia in humans after raw shellfish ingestion. It can also cause secondary septicemia when wounds are exposed to seawater (Carmona-Salido et al. 2021).

### 2.5. Aeromonadaceae

*Aeromonas* is another Gram-negative pathogen in fish with its infections remain asymptomatic until environmental stress and weakness. Both *Aeromonas* and *Vibrio* similarly infect fish, but *Aeromonas* is the prevalent species in freshwater fish while *Vibrio* species are found in freshwater as well as in brackish water, estuarine and marine. Both bacteria can be hazardous for human health (Boylan 2011). Fish play a fundamental role in *Aeromonas* transmission to humans (Abd-El-Malek 2017). The species reported with zoonotic potential include *A. hydrophila*, *A. caviae*, *A. jandaei*, *A. sorbia*, *A. salmonidae*, and *A. veroni*; among them, the most common pathogen is *A. hydrophila* (Khardori and Fainstein 1988; Vartian and Septimus 1990; Noga 2010; Boylan 2011). Nowadays, some fish pathogens such as *A. jandaei* and *A. veroni* can cause symptoms like *A. hydrophila* in fish. The *A. hydrophila* also causes opportunistic disease in weak fish as a secondary infection. Histopathological changes have been seen in organs such as the liver, kidney, gill, stomach, and spleen (AlYahya et al. 2018). Some clinical manifestations of *Aeromonas*-infected fish include petechiae in the skin and fins, skin ulcers, arrhythmias, anorexia, exophthalmia, and abdominal swelling (Agnew and Barnes 2007; Noga 2010). *Aeromonas* virulence factors include enzymes, enterotoxin, hemolysin, adhesin, flagella, lipopolysaccharide, secretory systems, and quorum sensing (Jin et al. 2020). Moreover, *Aeromonas* species can infect humans through ulcers or ingestion, although the infection is rare in humans. Some clinical effects in the individuals are muscle necrosis, cellulitis, and septicemia (Volpe et al. 2019). Clinical signs of the disease in humans include edema to swelling at the site of infection (Boylan 2011). Also, in humans, *Aeromonas* can cause bacteremia, respiratory infections, gastroenteritis, sepsis, urinary tract infections, and diarrhea. Multi-antibiotic resistance of *Aeromonas* is evidence of an emerging general health problem in humans and aquatic animals (Odeyemi and Ahmad 2017).

### 2.6. Pseudomonadaceae

*Pseudomonas* is an opportunistic Gram-negative bacillus as well as a cause of food poisoning (Yagoub 2009). As part of the fish’s natural microbiota, it poses a threat to fish in stressful situations (Algammal et al. 2020). However, with high genetic flexibility and adaptability to different environments, the bacteria can be found ubiquitously in environment, in various animals and humans (Benie et al. 2017). This motile bacterium has many virulence factors such as enzymes, pili, flagella, LPS, quorum sensing, and virulence factors that strengthen the inflammatory-invasive processes and the faster spread of bacteria. *P. fluorescent* has been known as an opportunistic pathogen in the aquatic environment, the digestive flora of healthy fish, and the natural microbiota of the aquatic environment and fish (Algammal et al. 2020). There is a report to find *P. septicemia* in freshwater, brackish, and marine (Guzman et al. 2013), but *Pseudomonas* septicemic agents in fish include *P. aeruginosa*, *P. anguilliseptica*, *P. putida*, and *P. fluorescens*. Clinical signs that have been observed include irregular hemorrhages on the body surface, exophthalmia, eye cloudiness, scales detachment, darkening of the skin, congested gills, ulceration, abdominal distention, and ascites. Most of the symptoms are caused by the bacterial extracellular enzymes and destructive toxins (Elham et al. 2017). Close human-animal contact is an important reason for transmitting the bacteria that are resistant to several antibiotics, such as *Pseudomonas*, which is a severe public health threat (Fernandes et al. 2018).

### 2.7. Enterobacteriaceae

The Enterobacteriaceae family is a fish microbiota that can cause human diseases. This family comprises species that cause a variety of human infections (Oliviera et al. 2017). Members of this family known as the zoonotic agents of fish include *Escherichia coli*, *Klebsiella* and *Salmonella* (Boylan 2011). These Gram-negative bacilli are found in aquatic environments and fish digestive tract (Boylan 2011; Oliviera et al. 2017). Infection detection of fish with *Enterobacteriaceae* families such as *E. coli*, *Klebsiella*, and *Salmonella* in Iran indicates their transmission to humans and the development of human infections (Faeed et al. 2005; Oliviera et al. 2017). The most common source of human infection with these bacteria is through open wounds, touch fish, or scratches that cause infection and inflammation at the entry point of bacteria and/or systemic infections (Smith 2011). On the other hand, there are human infections with some bacteria of this family through food sources; for example, infection with *S.* Typhimurium has been found due to the consumption of imported dried fish (Novotny et al. 2004; Bonyadian et al. 2014).

Infection of a variety of fish species with *E. coli* strains showed that fish is a new vector of this bacterium in water sources (Hansen et al. 2008). Fish can retain different strains of *E. coli* as flora and transfer them to other water sources (Guillen and Wrast 2010). Although *E. coli* is not a fish natural microbiota, it is often isolated from the fish digestive tract. Besides, infiltration of *E. coli* into other fish tissues such as gill, kidney, muscle, and bladder have been observed in contaminated environments (Barbosa et al. 2014). Such infections are related to the seasons, contact of a person with fish (infected fish are sometimes asymptomatic) and the contaminated environment, and a person’s immune status. *Escherichia coli* is one of the causes of zoonotic infections transmitted through fish or aquatic products. Most non-pathogenic strains become pathogenic, if they spread from the gut to other organs such as urinary tract or peritonium; but non-pathogenic strains cause diarrhea or food poisoning by producing toxins in fish (Haile and Getahun 2018). There are reports that some *E. coli* [enterotoxigenic (ETEC), enteropathogenic (EPEC)] are zoonotic agents isolated from different countries (Cardozo et al. 2018).

*Salmonella enterica* subspecies *enterica* is effective in the development of intestinal diseases through fish, aquaculture products, and water. *Salmonella* is not a common fish bacterium, and its occurrence depends on water quality and the aquatic environment. Fish can become asymptomatic hosts that keep bacteria on the surface of the body or in the gut. According to some studies, isolated *Salmonella* species of fish and water include *S.* Eastbourne, *S.* Give, *S.* Colindale, *S.* Bredeney, *S.* Poona, *S.* Schwarzengrund, and *S.* Llandoff. Human salmonellosis is dependent on the consumption of infected fish, the most common cause being *S.* Typhimurium and *S.* Enteritidis. Their virulence factors include proteins, secretory systems, intra-phagocyte proliferation, tissue viability, and intestinal lumen transfer. *Salmonella* persistence in fish digestion and its presence in feces is an important cause of environmental pollution and bacterial spread (Traoré et al. 2015). Consumption of *Salmonella*-infected fish causes symptoms such as gastroenteritis, abdominal cramps, fever, and bacteremia. *Salmonella*-infected smoked fish also can transmit bacteria to humans through the skin, gills, and intestines (Bibi et al. 2015). *Salmonella* infection also causes clinical complications such as sepsis, abdominal pain, diarrhea, and vomiting (Lehane and Rawlin 2000).

*Klebsiella pneumoniae* and *K. oxytoca* have been isolated from untreated water samples collected from dam, seawater, sediment, and intestinal contents of shrimps and freshwater fishes (Gundogan 2014; Gopi et al. 2016). Isolation and diagnosis of *K. pneumoniae* from farmed fish in India with clinical hemorrhagic complications near the tail and vacuolation and necrosis of hepatocytes have been reported. Due to the zoonotic nature of themulti-drug-resistance of *Klebsiella* spp. (*Klebsiella pneumoniae* complex), there are concerns about their transmission to humans (Das et al. 2018). Isolation of *Klebssiella* from the skin lesions of an ornamental fish, carp, revealed that the infectious process was due to poor hygiene by food processors (Oliveira et al. 2014). The manifestations of *Klebsiella*-infected fish are also due to the direct effect of endotoxin along with abnormal immunological responses (Diana and Manjulatha 2012).

Another Gram-negative bacterium that causes infection in freshwater and marine fish, is *Yersinia*. In recent decades, *Y. ruckeri*, the cause of severe septicemia, enteric redmouth (ERM) disease, has increased dramatically. The initial symptoms are similar to septicemia caused by *Aeromonas* and *Pseudomonas*, but fish have a darker body and less appetite. In addition, there are other symptoms such as exophthalmos, hemorrhages, reddening of mouth, swollen kidney, and spleen. The virulence of this bacterium depends on factors such as the secretory system, pili, enzymes, toxins, outer membrane proteins, flagella, iron acquisition system, heat sensitivity factor, and biofilm formation (Wrobel et al. 2019). Isolation of the bacterium in a wound infection of a person in contact with water hypothesized that the bacterium was zoonotic but still needed further evaluation (Keukeleire et al. 2014).

### 2.8. Hafniaceae

Hafniaceae is a family of motile, anaerobic, Gram-negative rod bacteria in the Order *Enterbacteriales* (Adeolu et al. 2016). Three genera of this family are *Hafnia*, *Edwardsiella* and *Obesumbacterium* (Adeolu et al. 2016). *Edwardsiella* are pathogenic to aquatic animals especially causing systemic fish disease called edwardsiellosis (Park et al. 2012; Yu et al. 2012). *Edwardsiella* has so far caused severe economic problems in the aquaculture industry, which has had a greater impact on fish at high ambient temperatures and higher concentrations of organic matter (Davies et al. 2018). Until 1980, *Edwardsiella* included only one species, *E. tarda*, but nowadays, five species are recognized, i.e. three older species: *E. tarda*, *E. hoshnae* and *E. ictaturi*, and recently two additional species, *E. piscicida* and *E. anguillarum* (previously classified as *E. tarda*) (Bujan et al. 2018). Except for *E. hoshnae*, other species are pathogenic in fish, and *E. tarda* is considered the major cause of human infections (Kerie et al. 2019). However, the new classification shows that *E. piscicida* has been more problematic in the aquaculture than *E. tarda* (Leung et al. 2019). So far, *Edwardsiella* infection can occur in over 20 fish species in Asia and Europe. Behavioral signs of infected fish include abnormal swimming, lateral movement and spiraling in the water column. For human, *E. tarda* is an opportunistic pathogen causing gastroenteritis; but extraintestinal edwardsiellosis may occur including wound and liver infections, cholecystitis, peritonitis, meningitis, myonecrosis, osteomyelitis, sepsis and bacteremia (Kerie et al. 2019). Although septicemia caused by *E. tarda* is a rare (< 5%) water-and food-derived infection of human but it can be fatal. People with immunodeficiency or underlying problems like diabetes and hepatobiliary illness are prone to the *E. tarda* created diseases. *Edwardsiella* species, especially *E. tarda* and *E. piscicida*, have an arsenal of virulence mechanisms such as types III and VI secretion systems (T3SS and T6SS), hemolysin EthA, translocation and assemble module (TAM) and antibiotic resistance genes (Wimalasena et al. 2018). More importantly, the bacteria can acquire mobile drug-resistant genes (plasmids), then transmit them to animal, human, and environmental microbiomes. Transmission of edwardsiellosis to humans is by swimming in contaminated water, consuming raw fish, contact with fish, and the state of the immune system. The entry and contamination of human cells are also through the attachment of bacteria to cells and the use of hemolysin and the secretion systems. *Edwardsiella* multiplies in phagocytes, which eventually spread to nearby cells. Because of the important role in creating antibiotic resistance, the bacteria should receive more attention in the coming decades (Leung et al. 2019).

### 2.9. Other bacteria associated with fish-derived zoonoses

Other zoonotic bacteria associated with fish consumption include *Staphylococcus*, *Listeria*, *Clostridium*, and *Campylobacter* (Novotny et al. 2004). The diagnosis of *Staphylococcus aureus*, especially methicillin-resistant *S. aureus* (MRSA), in food-producing animals like fish increases, and the presence of bacteria is considered an infection before or after harvest. Therefore, the focus on *Staphylococcus* is considered important for the fish food chain (Vaiyapuri et al. 2019).

Food handlers who have *S. aureus* in their skin and mucous membranes can act as a source of fish contamination (Obaidat et al. 2015). On the other hand, *S. aureus* enterotoxins cause gastroenteritis in human by eating fish and its products (Novotny et al. 2004). The heat-resistant *S. aureus* enterotoxins inflict public health concerns (Obaidat et al. 2015). Most studies on human infection with *S. aureus* have been with the consumption of contaminated fish, but recently *S. xylosus* has been reported as a primary pathogen that causes fish mortality. This emerging bacterium succeeds in defeating fish immunity causing exophthalmos, and fish death. Since raw fish is consumed in many countries, there is a risk of disease transmission to humans (Oh et al. 2019). Skin infection by *S. aureus* may lead to toxic shock syndrome (TSS) due to the bacterial TSST-1 toxin which is regarded as a super antigen that enters the blood stream and activates polyclonal T cells in peripheral blood causing massive release of pro-inflammatory cytokines (Rukkawattanakul et al. 2017).

According to the European Food Safety Authority (EFSA) data in 2016, *Listeria monocytogenes* are the utmost in fish and fishery products. The presence of this pathogen was confirmed in fish products (Skowron et al. 2019). *Listeria onocytogenes* is a Gram-positive bacterium that grows in a wide range of temperature including refrigerator and a variety of fresh and salty environments. The bacteria can survive in relatively low water conditions/activity, resistance to salt and freezing temperatures. Food was identified as the first source of infection of this bacterium in humans, and today it is a public health concern related to septicemia, meningitis, gastroenteritis, pneumonia, and abortion. *Listeria monocytogenes* is indigenous flora of surface water that can be found on the outer surface of fish, mucus/mucosa, intestines, stomachs, and gills of contaminated fish; therefore, the fish skin and fecal contents are the source of disease transmission (Jami et al. 2014). Elderly, pregnant women, and persons with chronic diseases/immunocompromised condition are high risk groups of human listeriosis (Lassen et al. 2016).

*Clostridium perfringens* and *Clostridium botulinum* (anaerobic rod-shaped spore-forming bacteria) are important food-derived pathogens caused by fish consumption. The bacteria are ubiquitous in soils, aquatic sediments, and natural anaerobic environments. They are associated with both fresh and canned fish (Novotny et al. 2004; Sabry et al. 2016). *Clostridium* is also present on the fish surface. *Clostridium perfringens* produces enterotoxins (CPE) from *cpe* gene, i.e. types A, C and D, that cause gastroenteritis in human (Freedman et al. 2016; Sabry et al. 2016). The toxins can also be absorbed from the intestine into blood circulation causing damage of tissues such as brain (Uzal et al. 2014). Spores of *Clostridium botulism* can remain in freshwater and sea sediments for decades. They are also found in the intestines of healthy fish (Espelund and Klaveness 2014). The bacteria produce botulinum toxins (types A-H) that inhibit acetylcholine release from synaptic vesicles at neuromuscular junctions causing flaccid paralysis (Collins and East 1998; Barash and Arnon 2014). Types A, B, E and F are toxic to human beings. Botulinum neurotoxin can sometimes be produced in the fish intestines. The botulinum toxins are relatively resistant to heat and require high heat treatment to destroy their toxicity. Therefore, consumption of improper food processing poses a risk to botulism which usually manifested early as diarrhea, vomiting, dizziness, dysphagia, bloating, and constipation (Rasetti-Escargueil et al. 2019).

*Campylobacter* is a common microorganism in the gastrointestinal tract of many animals and is a zoonotic agent (Facciola et al. 2017). Campylobacteriosis due to the fish product consumption is rare, but *Campylobacter jejuni* infection probably be acquired through the hands of a food handler or the work surface and drinking untreated water. *Campylobacter jejuni* and *C. coli* are the most important enteropathogens of this genus. The bacteria cause campylobacteriosis manifested as enteritis by using bacterial motility, intestinal cell adhesion and invasion, disturbing intracellular signaling, causing cell death, evasion of host immune system and acquisition of iron for their growth and survival (Epps et al. 2013). *Plesiomonas shigelloides* is a water-borne pathogen that has been isolated in freshwater fish (Nakajima et al. 1991). *Legionella pneumophila*, which causes legionnaires’ disease/pneumonia, is transmitted through water and aerosols, and was also isolated in a patient who worked at fish-market ( Novotny et al. 2004). *Yersinia ruckeri* causes yersiniosis or red mouth disease, a contagious bacteremia among salmonids, eels, goldfish, sole, sturgeon, trout, carps, and turbot. The disease is commonly detected due to exophthalmos and blood spots in the eye. The bacterium is found in fish populations throughout Europe, North and South America, Australia, and New Zealand (Tobback et al. 2007; Kumar et al. 2015; Carson et al. 2019).

## 3. Fish-derived parasites

The fish-derived parasitic tapeworms (e.g. *Dibothriocephalus latum*), roundworms (e.g. *Anisakis* spp.), and flukes (e.g. *Metagonimus yokogawaii*) are mainly transmitted to human beings via the consumption of improperly cooked or raw fish or fish products causing morbidity rather than mortality (Cong and Elsheikha 2021). There is plenty of literature on the significance of seafood within the global diet and the increasing health concerns of seafood-derived illnesses and associated parasitic diseases. Numerous edible fish are known to be hosts to numerous parasites (Shamsi 2019). Many of these parasites are transmissible to humans, and some, such as anisakidosis and gnathostomiasis, can be of serious concern to human health (Daengsvang 1981; Audicana et al. 2002; Herman and Chiodini 2009). Seafood, particularly fish products, is reported high on the list of food-derived illnesses (Huss et al. 2000). However, despite their abundance, parasites are usually overlooked in seafood safety discussions (Shamsi 2020). As a result, fish-derived parasites often go unrecognized and are responsible for several emerging zoonotic diseases (Dorny et al. 2009; Shamsi 2019). The standards of food inspection and protocols involved with checking for disease agents vary significantly between countries and are often inadequate and inconsistent (Williams et al. 2020). Even in developed countries, food safety regulations and import control for zoonotic parasitic diseases can be overlooked in food safety protocols (Shamsi 2016). Growing appetite for raw, undercooked, and exotic dishes (Shamsi and Sheorey 2018), along with climate changes, have been considered as main contributing factors for the increasing occurrence, geographical distribution, and frequency of zoonotic fish-related health problems (Chai et al. 2005; Lohmus and Bjorklund 2015). For example, it is estimated that 45 million people are currently infected with freshwater fish liver flukes, and at least 680 million people are at risk of infection with them (Saijuntha et al. 2021).

Among parasites of seafood, in particular, helminthic parasites are of significant concern, and due to their abundance and diversity in tropical aquatic ecosystems, their transmission to fish is a frequent occurrence (Chai et al. 2009; Ogbeibu et al. 2014). For example, 268 helminth species have been reported in 213 fish species in Vietnam (Nguyen et al. 2021). One reason is that the parasite lifecycle is trophic orientated, relying on the food web for host transmission (Polley and Thompson 2009). In addition, many edible teleost fish species are considered to be intermediate and sometimes paratenic hosts for helminthic parasites, which can result in the chances of helminthic infection increases with the size of the host (Marcogliese 2003). Normally, the number of parasites increases in correspondence to the host’s level within the food, with larger fish species harboring many parasites in them. However, plenty of zoonotic parasites may show no disease symptoms in the infected fish, so, detection is difficult, especially if the larvae are tiny and low-loaded (Lowry and Smith 2007; Shamsi and Suthar 2016).

According to Shamsi (2019), over 40 taxa of fish parasites are capable of causing human infection. While some are rarely found, others can be highly pathogenic and pose a serious risk to public health (Deardorff 1991). It has been estimated that helminthic parasites may put the health of more than half a billion people at risk (dos Santos and Howgate 2011). With global warming, this number is expected to be increased (Fiorenza et al. 2020). Fish-derived helminthic diseases may be associated with mild to severe allergic or gastrointestinal diseases such as indigestion, abdominal pain, and diarrhea, or may cause severe manifestations such as brain hemorrhage, hemiparesis and cancer (Germann et al. 2003; Sripa et al. 2011; Cong and Elsheikha 2021). The study conducted to estimate the occurrence of zoonotic parasites identified the presence of *Eustrongylides* sp., *Euclinostomum* sp. from Channidae fish and *Isoparorchis* sp. from Bagridae fish imported into Australia (Williams et al. 2022). Although freezing of imported edible fish inactivates the parasites, routine surveillance is required to prevent importation of zoonotic parasites (Williams et al. 2022).

### 3.1. Trematodes (flukes)

Several genera of trematodes (flukes) that belong to families *Opisthorchiidae* and *Heterophyidae* cause fish-derived zoonoses. Therefore, these parasites are included in the ParaFish Control project (Advanced Tools and Research Strategies for Parasite Control in European farmed fish) to control parasitic diseases in farms (Caffara et al. 2020). Examples of common flukes that infect fish/crabs are liver flukes, i.e. *Clonorchis sinensis*, *Opisthorchis viverrini*, and *Opisthorchis felineus* and lung flukes, e.g. *Paragonimus westermani*, and *P. heterotremus*. High liver fluke load and chronicity of the infection can cause inflammation and damage to the epithelial bile duct, leading to gastrointestinal problems and liver damage (Choi et al. 2004; Lin et al. 2005; Hung et al. 2015) and may lead to major clinical problems such as cholangitis, choledocholithiasis, pancreatitis, and cholangiocarcinoma (CCA) (Choi et al. 2004; Sripa et al. 2011; Boerlage et al. 2013). Paragonimiasis is lung fluke infection that human acquires after eating crab or crayfish (fresh water) harboring metacercariae of the flukes (Maleewong et al. 1992; Tantrawatpan et al. 2013). Fish-derived trematodiosis are highly prevalent in Asian countries and are a major cause of death in southeast Asia. *Opisthorchis viverrini*, the causative agent of CCA is highly prevalent in northeastern Thailand and Laos (Sripa et al. 2011; Fürst et al. 2012; Prueksapanich et al. 2018). Trematoda of zoonotic concern can be found in marine, brackish and freshwater fish (dos Santos and Howgate 2011), and the trematode infections usually occur after eating raw fish or shellfish in freshwater (Shamsi 2019). The zoonotic trematode metacercariae were detected in the freshwater fishes sampled from the Republic of Korea (Sohn et al. 2021). The metacercariae of *Clonorchis sinensis*, *Metagonimus* spp., *Centrocestus armatus*, *Echinostoma* spp., *Clinostomum complanatum*, *Opisthorchis viverrini*, and *Metorchis orientalis* were detected in the sampled fishes (Manivong et al. 2009; Sohn et al. 2021).

According to the WHO, zoonotic fish trematodes are listed among emerging infectious pathogens. In aquaculture systems, trematode infections and the transmission of contaminants in the environment can be risk factors for humans and other animals. According to reports of Clausen et al. (2012), the most identified species of trematodes in fish that are transmitted to humans include the following: *Clonorchis sinensis*, *Centrocestus formosanus*, *Haplorchis pumilio*, and *Haplorchis yokokawi* (Clausen et al. 2012). A rapid and cost-effective multiplex PCR is developed for simultaneous detection of Opisthorchiid and Heterophyid metacercariae in fish or fish products (Caffara et al. 2020). Such advanced diagnostic tools will help detect the infective stage (metacercariae) for human, which is difficult to detect visually in fish (Caffara et al. 2020). Domestic cats and dogs can act as the reservoir host of digenetic trematodes, particularly the zoonotic *Heterophyes heterophyes* and *O. viverrini.* Therefore, additional prevention and control measures have to be established that ensure continuous monitoring of fish zoonotic parasites in cats and dogs (Enes et al. 2010; El-Seify et al. 2021).

### 3.2. Cestodes (tapeworms)

Another common group of fish parasite is cestodes (tapeworms). Unlike trematodes, they can be quite large and may grow to 20 m in length. Some of the most well-known parasites belonging to this group include those in order *Diphyllobothriidae*, which can cause the disease called diphyllobothriosis (Scholz and Kuchta 2016). At least 14 of about 50 species of genus *Diphyllobothrium* have been reported to cause human infection (Jones 2015), with *D. dendriticum*, *D. nihonkaiense*, *D. latum*as well as *Adenocephalus pacificus*, and *Diplogonoporus balaenopterae* being the most pathogenic species (Anantawat et al. 2012). Diphyllobothriosis is usually a mild disease and is not life-threatening. Infected people are usually asymptomatic, but some may experience diarrhea, abdominal pain, anemia, weight loss, and vitamin B12 deficiency (Dick 2007; McConnaughey 2014). It is estimated that up to 20 million people worldwide are infected (Anantawat et al. 2012; Scholz and Kuchta 2016). However, except for in Japan and far Eastern Russia, human infection by tapeworms has declined globally.

### 3.3. Nematodes (round worms)

Human diseases from fish-derived nematodes are being recorded globally, but few of them represent potential emerging illnesses (Steffen et al. 2003; Butt et al. 2004; Nawa and Nakamura-Uchiyama 2004; Murrell and Fried 2007; Herman and Chiodini 2009; Eiras et al. 2018a). Human infections can occur, whereas squid or fish are eaten uncooked or improperly prepared, and diseases can have life-threatening effects, particularly for infected individuals. This illness is little understood in general, and as for countries of South America, that information has not yet been gathered. Nematode zoonotic species are found in both freshwater and marine fish of South America. As far as infections of humans are concerned, reports have been identified in a few nations, and their occurrence varies between countries. These infections are common in regions with strong fish-eating habits and are particularly numerous on the western coast of South America (Eiras et al. 2018a). Zoonotic nematodes belonging to the family Anisakidae were detected in the popular table fish *Chrysophrys auratus* sampled from Australian and New Zealand waters. *Anisakis pegreffii* identified in the *Chrysophrys auratus* will pose a significant threat to humans if served raw as sashimi or in sushi (Hossen et al. 2021). In addition, nematodes of zoonotic importance (*Contracaecum* spp., *Anisakis* spp., and *Hysterothylacium* spp.) were also detected in the edible fishes sampled in Australia (Suthar and Shamsi 2021).

Nematodes display little host specificity in their larval stages, which are infectious for humans. The larval stage of fish nematodes, even if found in the gastrointestinal tract of the fish, migrate through the gastrointestinal mucosa into the viscera and surrounding muscle tissues (Smith and Wootten 1978; Salikin et al. 2020) after the fish is dead. So, they can still pose a risk to human health. Some of the most common fish nematodes and concerns to human health are *Anisakis* spp., *Pseudoterranova* spp. causing anisakidosis and members of *Gnathostomatidae* causing gnathostomiasis. These nematodes are all globally or regionally considered as highly significant. The most common fish nematodes of the Anisakidae family include genera *Anisakis*, *Pseudoterranova*, and *Contracaecum* with worldwide distribution and are amongst the most reported larvae in marine parasites and are of considerable zoonotic importance (Borges et al. 2012; Shamsi and Suthar 2016; Bao et al. 2019; Safonova et al. 2021). Since their discovery in 1960, there has been an increased interest in the family *Anisakidae*. Several studies have been dedicated to increasing their awareness, improving their diagnostic techniques, and understanding numerous aspects of their pathogenicity and biology (Shamsi 2014). Anisakiasis or anisakiosis is the term used to indicate the parasitic infection caused by nematodes of the genus *Anisakis* in humans. It is caused by the parasite third-stage larvae (L3) (Adroher-Auroux and Benítez-Rodríguez 2020). Anisakidosis is the disease caused by any members of the family Anisakidae, while anisakiasis is created by members of the genus *Anisakis*. Members of *Anisakis simplex sensulato* are the common causative agents for anisakidosis. Other nematodes, include, *Pseudoterranova decipiens*, *A. physeteri* and *Contracaecum* spp. (Audicana and Kennedy 2008; Eiras et al. 2018b). *Anisakis* larvae have been previously detected in fish products such as fish steaks, frozen fish fillets, fish fingers, and cod fillets (Ramos 2020). The presence of *Anisakis* nematodes was investigated in the smoked wild sockeye salmon (*Oncorhynchus nerka*) and farmed Atlantic salmon (*Salmo salar*) products (Pardo González et al. 2020). Although the samples collected from smoked wild sockeye salmon were tested positive (10 out of 13) for *Anisakis simplex s.s.* larvae, no parasites were detected from the farmed Atlantic salmon samples indicating negligible risk in farmed fish (Pardo González et al. 2020). Different strategies have to be implemented to prevent the entry of *Anisakis* and other parasites into the fish farms. This includes the freezing of trash fish used for feeding farmed fish and the reinforcement of water entry points using nets to prevent the entry of wild fish (Ramos 2020). The risk of anisakiasis can be further reduced by subjecting the entire raw fish to thermal treatment (>60 °C, >1 min or − 20 °C, >24 h) prior to consumption (Pardo González et al. 2020). Considering the potential risk of transmitting zoonotic fish parasites via raw or undercooked fish and fish products, European Union (EU) made freezing treatment mandatory for fish products [Regulation No. 1276/2011 modified the Annex III of Regulation (EC) No 853/2004] (Fioravanti et al. 2021).

Humans are considered accidental hosts within the anisakid life-cycle, as parasite development is arrested (hypobiosis) within the human gastrointestinal tract (Anderson 2000; Aibinu et al. 2019). Anisakidosis is usually caused by live larvae, and symptoms of human gastroenteritis result from larvae entering the gastric or intestinal mucosa (Audicana and Kennedy 2008; Ramanan et al. 2013); however, dead anisakids can cause disease as well (Audicana et al. 2002). Anisakidosis usually is presented with gastrointestinal symptoms and often mimics food poisoning. Symptoms can vary depending on the location of the parasite in humans and the histopathology of the associated lesions (Chai et al. 2005). Symptoms are variable to the individual patient and can last for days up to months. Generally, symptoms subside after the parasite is passed naturally (regurgitation or excretion) from the body (Shamsi and Butcher 2011) or removed surgically (Shimamura et al. 2016). *Anisakis-*associated hypersensitivity is an important concern. Sensitive patients show high sensitivity as even very small doses of exposure to dead *A. simplex* material properly cooked may cause potentially lethal and rapid-onset anaphylactic to rather chronically disabling conditions (Aibinu et al. 2019). Although the disease has been reported worldwide, it is more common in Japan and in Europe (Rahmati et al. 2020), and it is believed to be significantly underreported and/or misdiagnosed due to non-specific symptoms in patients (Shamsi and Butcher 2011) and limited availability of diagnostic tests. For example, even in Japan where the disease is well known, it has been shown that 60% of situations were misdiagnosed as acute abdomen infection, appendicitis, ileitis, cholecystitis, gastric and pancreas cancer, tuberculous peritonitis and diverticulitis (Yokogawa and Yoshimura 1967; Valle et al. 2012; Nieuwenhuizen 2016).

*Gnathostomatidae* is another highly important nematode infection from eating raw or undercooked dishes such as sushi, ceviche containing fresh- and brackish-water fish species, as well as other fresh water animals (amphibians, eels). The parasites cause a disease known as gnathostomiasis, from the ingestion of infective larvae (L3) of the family *Gnathostomatidae* including *Gnathostoma spinigerum*, *G. doloresi*, *G. hispidum*, *G. binucleatum*, *G. nipponicum* and *G. malaysiae* as well as *Echinocephalus* sp. (Daengsvang 1981; Anantawat et al. 2012; Pinheiro et al. 2017; Shamsi et al. 2021). Apart from hypo-allergic responses, the clinical symptoms of gnathostomiasis are similar to *A. simplex* but are normally more severe (Anantawat et al. 2012) and include nausea, abdominal pain, and vomiting, which usually develop 24–48 hours after transmission. The parasite infective larva migrates through the subcutaneous tissues causing typical inflammatory migratory swellings and may penetrate throughout the skin, lungs, eyes, ears, gastrointestinal and genitourinary systems and may results in brain hemorrhage, paresis or fatality if it occurs in the nervous system. The disease has been reported in Japan and Southeast Asia, particularly throughout Thailand, Vietnam, Lao-PDR, Myanmar as well as Central and South America, Latin America, China, India, and in travelers returning from these regions (Herman and Chiodini 2009; Liu et al. 2020; Sawadpanich et al. 2021). Most cases of the gnathostomiasis have been assigned to *Gnathostoma spinigerum*. Other species, i.e. *G. hispidum*, *G. doloresi*, *G. binucleatum*, and *G. nipponicum* are also of zoonotic significance (Shamsi et al. 2021).

## 4. Viruses that can be transmitted from fisheries to cause human diseases

Acute gastroenterritis caused by Noroviruses (NoV) in the form of either sporadic cases or outbreak through consuming ready-to-eat fishery products and shellfish contaminated with feces is gaining importance as the emerging food-derived illness of a public health concern that impacts economic loss worldwide (Pavoni et al. 2013; Li et al. 2014; Kittigul et al. 2016; Marsh et al. 2018). Noroviruses are non-enveloped positive-sense single stranded-RNA viruses that belong to *Caliciviridae* family. The genus *Norovirus* comprises of only one species called *Norwalk virus*.

Noroviruses are classified further into seven genogroups (GI-GVII) (Atmar et al. 2019). Most genogroups that infect human are genogroups GI and GII (Vinjé et al. 2000). Clinical manifestations of the NoV-mediated gastroenteritis include nausea, vomiting, watery diarrhea, and abdominal pain. Lethargy, weakness, muscle aches, headaches, and low-grade fevers and loss of taste may occur. Symptoms usually manifest about 12–48 hours after consuming the contaminated food but in most cases the disease is self-limited, except for people with immunocompromised conditions that may acquire long-term infection with the virus-associated enteropathy and malabsorption (Center of Disease Control and Prevention (CDC) 2016).

Consuming fresh and frozen food including fish, bivalves and water contaminated with hepatitis A virus (HAV) leads to hepatitis (inflammation of liver) manifested as fatigue, nausea, vomiting, diarrhea, jaundice, dark urine, fever, abdominal pain, arthralgias, myalgias, which may last for few weeks or several months. But in rare cases, liver failure or even death may occur, particularly in elderly and people with chronic liver disease.

## 5. Fungal zoonotic agents

Fungi are known as non-photosynthetic microorganisms. They usually live as saprophytes in dead organic matter and soil or as parasites of animals, plants, and humans. Of the 1.5 million identified fungal species, only 300 are known to be pathogenic to humans (Centers for Disease Control and Prevention (CDC) 2017). Common fungi in the environment often cause fungal diseases. Zoonotic fungi, which can transmit naturally between animals and humans, can sometimes lead to considerable public health problems. Nevertheless, insufficient attention to zoonotic fungi in international public health efforts has led to a decline in the development of prevention and control strategies. The following are two groups of fish zoonotic fungi.

### 5.1. Basidiobolomycosis

Basidiobolomycosis caused by *Basidiobolus ranarum* is a rare fungal infection (Shreef et al. 2017). This causative agent is found as a wide-spread environmental saprophyte isolated from putrefying plant materials, foodstuff, and leaves of deciduous trees, fruits, and soil.

*Basidiobolus ranarum* belongs to the class *Zygomycetes*, order *Entomophthorales* and the phylum *Zygomycota* (Anaparthy and Deepika 2014). It has been hypothesized that the mode of acquisition of *Basidiobolus ranarum* infection is through the skin following scratch, cut, or bite of insects. The other accessible sites of this fungal infection include the thigh, buttock, and perineum (Mugerwa 1976; Singh et al. 2008). It has also been reported in the gastrointestinal tracts of animals such as amphibians (e.g. toads and frogs), reptiles (e.g. geckos and garden lizards), and fish as well as mammals (e.g. insectivorous bats, dogs, horses, and humans) (Okafor et al. 1984; Zahari et al. 1990; Gugnani 1999; Bigliazzi et al. 2004; El-Shabrawi and Kamal 2011). The disease usually occurs as a subcutaneous and gastrointestinal infection (Mantadakis and Samonis 2009; Ageel et al. 2017). The disease has been observed in tropical regions, including Asia, Africa, Europe, South America, and the USA (Bittencourt et al. 1979). The spores of this fungus grow slowly after penetrating the body through a scratch in the skin, which can produce an enlarged hard node beneath the skin, especially in the arms and legs (Okafor et al. 1984). Ingesting the soil or animal feces-contaminated food is another way of transmitting this zoonotic pathogen (Shreef et al. 2017). If left untreated, it can lead to the patient’s death by penetrating deeper tissues and infecting pivotal organs such as the brain.

In 1964, the first case of gastrointestinal basidiobolomycosis (GIB) was reported in a child boy (Rabie et al. 2011). In addition, a 3-year-old girl who was infected with *B. ranarum* experienced ulcerations and painful swelling on the right leg for one and six months, respectively. In histopathological sectioning, dermal granulomatous inflammatory infiltrations with broad and septate fungal hyphae and yeast-like structures were observed (Sackey et al. 2017). Similar cases have been reported in infants with painless swellings on the leg and progressive ulcers toward underlying muscles (Mendiratta et al. 2012; Anaparthy and Deepika 2014).

Subsequently, many new cases were reported in various countries, such as Iran, the USA, Saudi Arabia, and Kuwait. It has been observed that levels of several cytokines including Th2-type cytokines (IL-4, IL-10), and proinflammatory cytokine, TNF-α are elevated following *B. ranarum* infection. IgM and IgG specific antibodies are produced against the locally infecting fungi, and this could be applied as a diagnostic measure (Khan et al. 2001).

### 5.2. Sporotrichosis

The fungal disease named sporotrichosis is caused by the *Sporothrix schenckii*, a dimorphic fungus that is particularly prevalent in tropical and subtropical regions in Mexico, Peru, Brazil, Uruguay, Japan, and India (Barros et al. 2011). The fungus lives naturally as saprophyte on living and putrefying vegetation, soil, and animal feces (Kenyon et al. 1984; Kwon-Chung and Bennett 1992). Thus, infection generally occurs through fungal-contaminated plants, soil, and organic matter. Hunting, fishing, gardening, farming, and other similar activities facilitate the fungus transmission (Rippon 1988; Barros et al. 2011). In addition, reports confirm the transmission of the fungus through the bites of insects and animal scratches such as squirrels, cats, dogs, horses, rodents, and birds (Kwon-Chung and Bennett 1992; Saravanakumar et al. 1996; Fleury et al. 2001). *Sporothrixs chenckii* has also been isolated from insects directly contacted to the fungus (Kwon-Chung and Bennett 1992) and aquatic animals, primarily fish and dolphins (Migaki et al. 1978; Haddad et al. 2002). In some rural areas, sporotrichosis has become an endemic disease that affects a specific group of workers such as woodcutters and farmers.

Overall, since the fungus exists as a free-living microorganism in the environment, all age and gender groups will be susceptible to this fungal infection (Sharma et al. 1990; da Rosa et al. 2005; Mahajan et al. 2005). In a report from a rural area of São Paulo state, fisherman’s finger injury with a fungus-infected dorsal fin spine of a fish (*Tilapia* sp.) led to ulceration, edema, pain, and purulent discharge in the affected area (Haddad et al. 2002). The disease occurs in localized forms in about 98% of cases (Bargman 1983). According to a published report in 1978, the Fishers in Guatemala were recognized as an endemic focus for sporotrichosis, and the isolation of the fungus from the fish in this area reinforces the view that a wound of the hand in this case likely became polluted with *S. schenckii* inoculum on the fish surface (Mayorga et al. 1978). It seems less likely that the inoculation of *S. schenckii* originated from other sources that previously on the patient’s hand surface area. There are three main clinical types of sporotrichosis: 1) lymphocutaneous sporotrichosis, 2) fixed-cutaneous sporotrichosis, and 3) multifocal/disseminated-cutaneous sporotrichosis. Systemic sporotrichosis is caused by the hematogenous spread of the fungus from the primary site of inoculation, lymph nodes, or patients with respiratory problems (Itoh et al. 1986; da Rosa et al. 2005; Bonifaz et al. 2007). The common forms of sporotrichosis that would be treated easily, are cutaneous and subcutaneous. Infectious Diseases Society of America recommends the use of oral therapy with itraconazole as a first-line treatment of subcutaneous sporotrichosis (Mahajan 2014).

## 6. Prevention and control

Microbial agents in fish can increase public health problems, so educating the public about microorganisms and the dangers of eating raw or undercooked fish is important. Quality control measures and regular monitoring of consumed fish are required. This enables rapid and effective control of diseases and provides the necessary information for preventing and treating aquatic zoonotic agents (Bibi et al. 2015).

Controlling fish zoonotic agents is challenging because fishes are raised in a system where output is based on natural environmental conditions. Most fish diseases are caused by the degradation of the aquatic environment, and environment also represents a significant factor in affecting fish health. As a result, interdisciplinary strategies encompassing the information to the potential pathogens for fish, elements of fish biology, and a good understanding of environmental factors will enable the application of appropriate measures to prevent and control diseases (Toranzo et al. 2005). Cleaning and sterilizing ponds effectively lowers the number of intermediate hosts of some nematode species to disrupt the lifecycle. Ponds that have not been cleaned and sterilized before refilling are at increased risk of retaining large numbers of intermediate hosts (Clausen et al. 2012; Hedegaard et al. 2012; Tran et al. 2019).

Determinants of fish-derived disease in populations can vary from the geographical locality and access to fresh seafood to sanitation, fish-handling techniques, and diets. Personal and social behavior is also very important (Deardorff 1991). Unlike many other diseases, fish-derived diseases are not limited to middle and low-income countries (Chai et al. 2005). Factors like growing international markets, consumer demand, rectified transportation systems, and demographic changes have led to fish-derived diseases being important in developed countries (Shamsi 2016). Some actions can be taken during harvesting, storage, processing, and post-processing that can be useful to minimize the risk posed by zoonotic pathogens. The application of various programs by government authorities and the seafood industry, including good manufacturing practices (GMP’s) and HACCP systems can contribute to the control of the risks posed by zoonotic fish-derived helminths (Adams et al. 1997). However, antibiotics are a way to control some zoonotic factors and antibiotic treatment is abundant in bacterial zoonotic pathogens (Durborow 1999; Shin and Park 2018); people related to fish must be aware of zoonotic diseases and ways to prevent. The prevention could be the best way to reduce the risks of these zoonotic infections; in particular, it is impractical to have no contact with water and fish in the aquaculture system (Smith 2011). In the case of food-derived zoonoses, multidrug-resistant animal pathogens are transmitted to humans through the consumption of contaminated food. To solve this fundamental problem, it is important to monitor multidrug-resistant microbes in humans and animals as the uniform functioning of communities. In addition, it requires strong assistance between physicians, veterinarians and environmental experts (Chowdhury et al. 2021).

**Figure 2.**
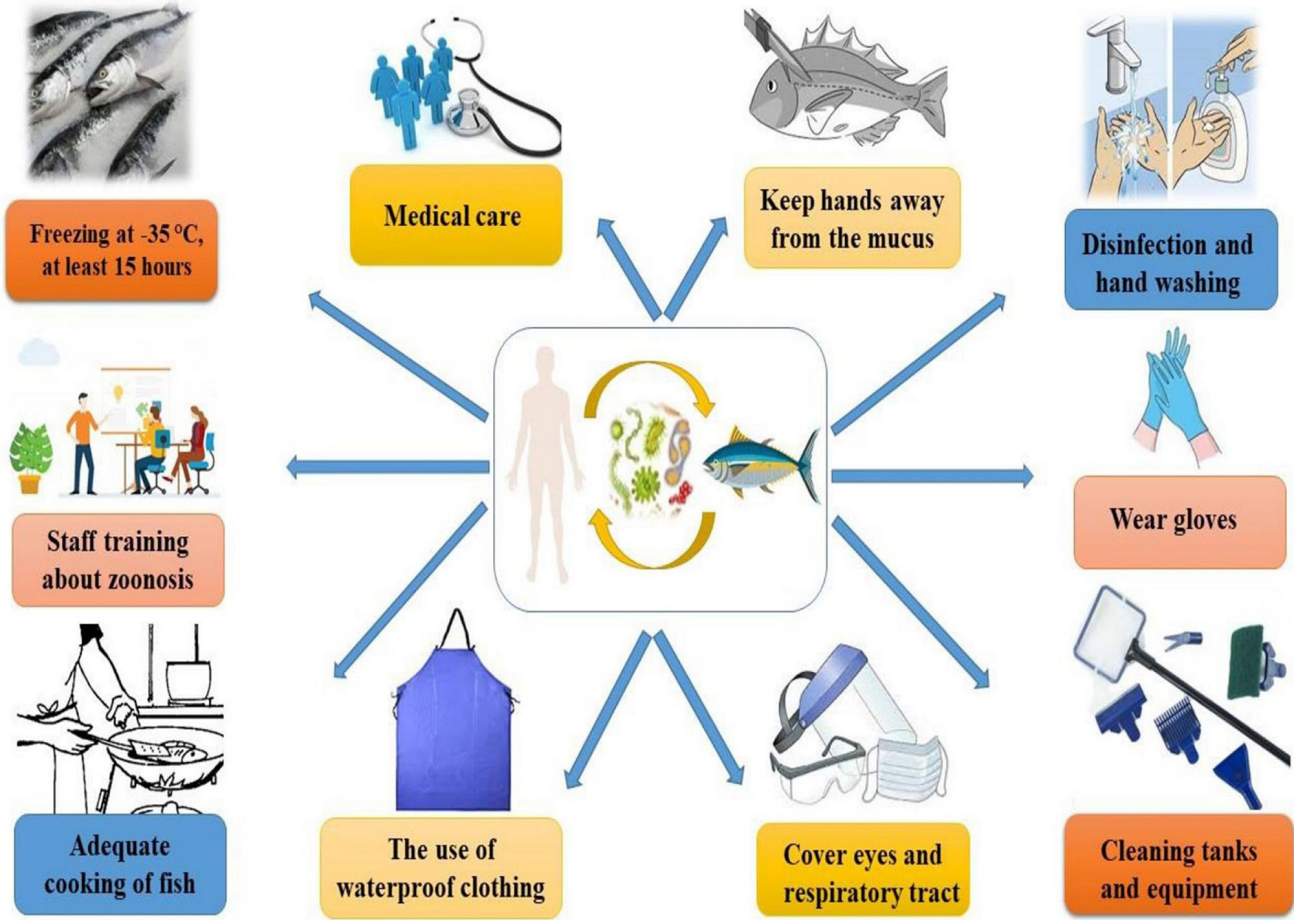
A schematic representation of possible methods and ways for control and prevention of zoonotic diseases.

Wearing disposable gloves and keeping skin away from the fish mucus is important. Consulting with the physician is vital even if nonspecific symptoms occur. The most effective way, especially after direct contact with fish and water, is frequent handwashing. Moreover, it is important to avoid eating and drinking before handwashing. Transmission of zoonotic diseases is also through direct or indirect contact with vectors, insects, and contamination with inanimate objects, swallowing, and inhalation (Boylan 2011). Appropriate methods of dealing with fishing vessels and technological factories are required to prevent contamination with fish parasites. On the other hand, cooking fish at 62 °C for 15 seconds is enough to kill parasites (but may not enough to detoxify some bacterial toxins). If there are parasites in fish, the following options can do for parasite removal and fish testing: complete cooking and informing fish sellers to check the remaining fish (Seafood Health Facts (SHF) 2020). One of the most effective means of reducing risk is by freezing or heat-inactivation (Ahuir-Baraja et al. 2021). Consequently, some necessary strategies must be considered for control, prevention, and monitoring of zoonotic pathogens, as shown in Figure 2.

Aquaculture systems differ in size and structure from small home aquariums to large hectare ponds, but they always include nutrient-rich water that encourages bacterial growth. Therefore, multiple studies on effective chemical disinfecting of contaminated environments have been done; contact duration, correct and safe handling of disinfectants, and precise disinfectant dosage should all be addressed to effectively prevention of fish zoonoses. Interestingly, simple desiccations/drying have also been considered an effective disinfection technique for controlling zoonotic bacterial diseases (Chen 1995; Murrell 2002). Although freezing of imported edible fish inactivates the parasites, not every fish captured undergoes freezing (Williams et al. 2022). The increasing preference of consumers toward ready-to-eat seafood items such as raw fish flesh slices can contribute to zoonotic transmission (You et al. 2021). Such cultural practices will prevent the successful implementation of prevention and control measures to counter the emergence of zoonotic disease outbreaks. Ready-to-eat raw fish products such as sushi and sashimi are classified as a biological hazard. Therefore, governments should develop strict regulations that monitor the safety and quality of fish used for such purposes (Lehel et al. 2021). Furthermore, the sudden growth of the freshwater ornamental fish industry should also be considered as a major human-fish interface where there can be potential transmission of zoonotic diseases. The reports of *Mycobacterium* sp. in freshwater ornamental fishes indicate the severity of this issue (Phillips Savage et al. 2022).

**Figure 3.**
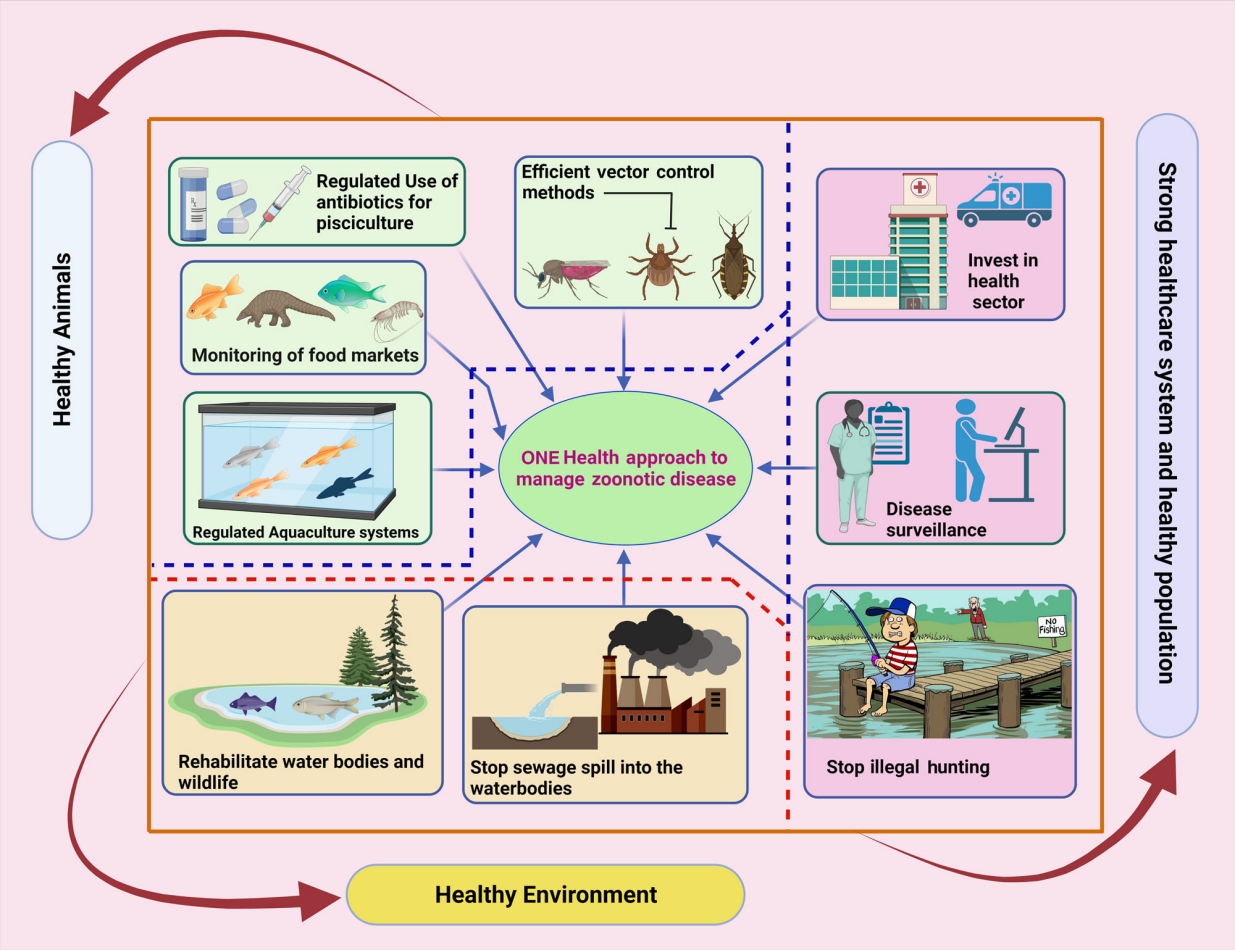
One Health (OH) approach in managing fish zoonotic disease. Augmentation of healthy animals with a healthy environment and healthy population along with strong health care infrastructure can prevent any potential fish zoonotic outbreaks. The figure was created with BioRender.com.

Veterinarians and fish handlers should always protect themselves by limiting their exposure to water when they have open wounds or abrasions. Disposable gloves can safeguard fish handlers throughout various operations, including contact with fish mucus, tissue, or waste of fish products. When water contact is inevitable, gels, tissue glue, and topical ointments, including triple antibiotic and silver sulfadiazine, can be applied on the surface wounds; nevertheless, disposable gloves are still suggested. Deep penetration injuries should be washed with normal water or saline water as soon as possible after the injury occurs and the wound is adequately disinfected with agents such as hydrogen peroxide, alcohol, betadine, or chlorhexidine. Severe bruises pose a greater hazard and should be treated immediately. Veterinarians have a responsibility to enlighten customers and lead by example in terms of correct PPE use. When working with fish, clients should be warned about zoonoses without exaggerating the hazards. Knowledgeable clients can provide a detailed history to their physician if a probable fish-derived zoonosis is detected. To instruct and assist in managing fish-derived zoonoses, veterinarians must interact with customers, employees, and hospitals (Grant and Olsen 1999; Boylan 2011). One Health (OH) approach has gained importance in managing the zoonotic fish diseases and needs to be strengthened and widely implemented (Figure 3). It is worthwhile to increase the deep connection and participation of stakeholders in solving One Health challenges for seafood safety (Shamsi 2019).

In addition to that, WHO reports globalization and the movement of people, animals, and goods across borders have led to the spread of zoonotic diseases. In addition, the lack of public health in remote communities, the lack of proper systems for transporting samples, and limited laboratory facilities for early diagnosis of the disease have led to the further dispersion of the pathogens. According to WHO reports, the basic challenges in limiting of One Health system and managing zoonotic diseases are organizational, control, interruption of transmission, and diagnosis/detection. Hence, the most important guidelines in this regard are effective cooperation between human and animal health officials, improving early detection of disease and pathogens, promoting infection management, as well as controlling vectors and rodents (World Health Organization (WHO) 2021). Furthermore, it is crucial to monitor the ‘One Health’ methodology while training university students, research centers, research groups, and international agencies to incorporate interdisciplinary and inter-sectoral organizations to regulate and prevent zoonoses. One Health (OH) is an interactive, multidisciplinary, integrated, and multi-sectoral approach that works at various levels (local, regional, national and global) to achieve the desired health outcomes by knowing of the relationships between people, animals, and plants and their shared environment. It is important to note that the success of One Health requires international participation between health and treatment systems (Aggarwal and Ramachandran 2020). Integrating regulated farming of fishes with several countermeasures such as reducing the water pollution and upgraded disease surveillance system along with various other measures can control the fish-derived zoonotic diseases or any potential disease outbreak (Marbán-Castro et al. 2019; Aggarwal and Ramachandran 2020).

## 7. Conclusion and future prospects

Fish are host to various pathogens, some of which are zoonotic and can cause human infection. With an increasing demand for and consumption of seafood, zoonotic agents have become a critical concern for the global health sector and fishing industries, which has stimulated an increase in marine zoonotic research. However, the biodiversity, ecology, occurrence, and distribution of fish-derived pathogens are still inadequate, particularly about the parasites. The lack of data on the occurrence and prevalence of zoonotic factors has led to the current undertaking of this review. Studies on the full range of hosts and the occurrence of geographical distribution, and the consequences of seasonality on the prevalence of infection have yet to be completed. Furthermore, a clearer understanding of the morphological identification of pathogens needs to be determined to better our knowledge of their occurrence within their environment and improve our awareness within the food industry, biosecurity, and medical practices.

The majority of the fish-derived parasitic tapeworms, roundworms, and flukes are mainly transmitted to human beings via the consumption of improperly cooked or raw fish (sashimi and sushi) or fish products (fish steaks, frozen fish fillets, fish fingers, and cod fillets). The risk of fish-derived parasitic tapeworms, roundworms, and flukes can be reduced by subjecting raw fish to thermal or freezing treatment before consumption. In addition, preventive measures should be established in the farm premises to prevent the entry of wild fish. Advanced molecular diagnostic techniques should be developed to detect fish-derived zoonotic agents specifically. This will ensure easy and cost-effective surveillance of zoonotic pathogens in freshwater, cultured, marine, and ornamental fishes. Consequently, fish as a food source is economically valuable, but the presence of some probably zoonotic pathogens has led to the spread of aquatic infections in humans. Therefore, having sufficient information about these binaries and teaching control and prevention methods is vital in public health and should be considered as an important aspect for human societies. In addition, integrating the One Health approach by augmenting various control measures can be a sustainable and reliable strategy to manage any fish zoonotic or any other potential disease outbreak. Following the One Health approach can be challenging due to the involvement of various factors and complexity associated with them. Still, it is certain is that this can prevent any future zoonotic diseases or an outbreak in the community (Zorriehzahra and Talebi 2021).

## References

[CIT0001] Abd-El-Malek A. 2017. Incidence and virulence characteristics of *Aeromonas* spp. in fish. Vet World. 10(1):34–37. 28246446 10.14202/vetworld.2017.34-37 PMC5301177

[CIT0002] Adams A, Murrell K, Cross J. 1997. Parasites of fish and risks to public health. Rev Sci Tech. 16(2):652–660. 9501379 10.20506/rst.16.2.1059

[CIT0003] Adeolu M, Alnajar S, Naushad S, Gupta RS. 2016. Genome-based phylogeny and taxonomy of the ‘Enterobacteriales’: proposal for Enterobacterales ord. nov. divided into the families Enterobacteriaceae, Erwiniaceae fam. nov., Pectobacteriaceae fam. nov., Yersiniaceae fam. nov., Hafniaceae fam. nov., Morganellaceae fam. nov., and Budviciaceae fam. nov. Int J Syst Evol Microbiol. 66(12):5575–5599. 27620848 10.1099/ijsem.0.001485

[CIT0004] Adroher-Auroux FJ, Benítez-Rodríguez R. 2020. Anisakiasis and *Anisakis*: an underdiagnosed emerging disease and its main etiological agents. Res Vet Sci. 132:535–545. 32828066 10.1016/j.rvsc.2020.08.003

[CIT0005] Ageel HI, Arishi HM, Kamli AA, Hussein MH, Bhavanarushi S. 2017. Unusual presentation of gastrointestinal Basidiobolomycosis in a 7-year-old child case report. Am J Med Case Rep. 5(5):131–134.

[CIT0006] Aggarwal D, Ramachandran A. 2020. One health approach to address zoonotic diseases. Indian J Commun Med. 45(Suppl 1):S6–S8. 10.4103/ijcm.IJCM_398_19 PMC7232973 32476732

[CIT0007] Agnew W, Barnes A. 2007. *Streptococcus iniae*: an aquatic pathogen of global veterinary significance and a challenging candidate for reliable vaccination. Vet Microbiol. 122(1-2):1–15. 17418985 10.1016/j.vetmic.2007.03.002

[CIT0008] Ahmadi Balootaki M, Amin A, Haghparasti F, Rokhbakhsh-Zamin F. 2017. Isolation and detection of *Erysipelothrix rhusiopathiae* and its distribution in humans and animals by phenotypical and molecular methods in Ahvaz-Iran in 2015. Iran J Med Sci. 42(4):377–383. 28761204 PMC5523045

[CIT0009] Ahuir-Baraja AE, Llobat L, Garijo MM. 2021. Effectiveness of Gutting Blue Whiting (*Micromesistius poutassou*, Risso, 1827), in Spanish supermarkets as an anisakidosis safety measure. Foods. 10(4):862. 33921070 10.3390/foods10040862 PMC8071357

[CIT0010] Aibinu IE, Smooker PM, Lopata AL. 2019. *Anisakis* nematodes in fish and shellfish- from infection to allergies. Int J Parasitol Parasites Wildl. 9:384–393. 31338296 10.1016/j.ijppaw.2019.04.007 PMC6626974

[CIT0011] Algammal AM, Mabrok M, Sivaramasamy E, Youssef FM, Atwa MH, El-Kholy AW, Hetta HF, Hozzein WN. 2020. Emerging MDR‑*Pseudomonas aeruginosa* in fish commonly harbor oprL and toxA virulence genes and blaTEM, blaCTX‑M, and tetA antibiotic‑resistance genes. Sci Rep. 10(1):15961. 32994450 10.1038/s41598-020-72264-4 PMC7524749

[CIT0012] AlYahya S, Ameen F, Al-Niaeem K, Al-Sa’adi BA, Hadi S, Mostafa AA. 2018. Histopathological studies of experimental *Aeromonas hydrophila* infection in blue tilapia, *Oreochromis aureus*. Saudi J Biol Sci. 25(1):182–185. 29379378 10.1016/j.sjbs.2017.10.019 PMC5775099

[CIT0013] Anantawat S, Liermeier A, McLeod C, Sumner J. 2012. Semi-quantitative risk assessment of harmful parasites in Australian Finfish. South Australia Research and Development Institute, 2012.

[CIT0014] Anaparthy UR, Deepika G. 2014. A case of subcutaneous zygomycosis. Indian Dermatol Online J. 5(1):51–54. 24616857 10.4103/2229-5178.126033 PMC3937489

[CIT0015] Anda P, Segura del Pozo J, Díaz García JM, Escudero R, García Peña FJ, López Velasco MC, Sellek RE, Jiménez Chillarón MR, Sánchez Serrano LP, Martínez Navarro JF. 2001. Waterborne outbreak of tularemia associated with crayfish fishing. Emerg Infect Dis. 7(3 Suppl):575–582. 11485678 10.3201/eid0707.010740 PMC2631832

[CIT0016] Anderson RC. 2000. Nematode parasites of vertebrates: their development and transmission. 2nd ed. CAB International. p. 672.

[CIT0017] Atmar RL, Baehner F, Cramer JP, Lloyd E, Sherwood J, Borkowski A, Mendelman PM, NOR-201 Study Group. 2019. Persistence of antibodies to 2 virus-like particle Norovirus vaccine candidate formulations in healthy adults: 1-year follow-up with memory probe vaccination. J Infect Dis. 220(4):603–614. 31001633 10.1093/infdis/jiz170

[CIT0018] Audicana MT, Ansotegui IJ, de Corres LF, Kennedy MW. 2002. *Anisakis simplex*: dangerous-dead and alive? Trends Parasitol. 18(1):20–25. 11850010 10.1016/s1471-4922(01)02152-3

[CIT0019] Audicana MT, Kennedy MW. 2008. *Anisakis simplex:* from obscure infectious worm to inducer of immune hypersensitivity. Clin Microbiol Rev. 21(2):360–379. 18400801 10.1128/CMR.00012-07 PMC2292572

[CIT0020] Austin B. 2010. Vibrios as causal agents of zoonoses. Vet Microbiol. 140(3-4):310–317. 19342185 10.1016/j.vetmic.2009.03.015

[CIT0021] Baiano JCF, Barnes AC. 2009. Towards control of *Streptococcus iniae*. Emerg Infect Dis. 15(12):1891–1899. 19961667 10.3201/eid1512.090232 PMC3044519

[CIT0022] Bao M, Pierce GJ, Strachan NJC, Pascual S, González-Muñoz M, Levsen A. 2019. Human health, legislative and socioeconomic issues caused by the fish-derived zoonotic parasite Anisakis: challenges in risk assessment. Trends Food Sci Technol. 86:298–310.

[CIT0023] Barash JR, Arnon SS. 2014. A novel strain of *Clostridium botulinum* that produces type B and type H botulinum toxins. J Infect Dis. 209(2):183–191. 24106296 10.1093/infdis/jit449

[CIT0024] Barbosa MMC, Pinto FD, Ribeiro LF, Guriz CSL, Ferraudo AS, Maluta RP, Rigobelo EC, Ávila FA, Amaral LA. 2014. Serology and patterns of antimicrobial susceptibility in *Escherichia coli* isolates from pay-to-fish ponds. Arq Inst Biol. 81(1):43–48. São Paulo

[CIT0025] Bargman H. 1983. Sporotrichosis of the skin with spontaneous cure. J Am Acad Dermatol. 8(2):261–262. 6826823 10.1016/s0190-9622(83)80196-0

[CIT0026] Barkham T, Zadoks RN, Azmai MNA, Baker S, Bich VTN, Chalker V, Chau ML, Dance D, Deepak RN, van Doorn HR, et al. 2019. One hypervirulent clone, sequence type 283, accounts for a large proportion of invasive *Streptococcus agalactiae* isolated from humans and diseased tilapia in Southeast Asia. PLoS Negl Trop Dis. 13(6):e0007421. 31246981 10.1371/journal.pntd.0007421 PMC6597049

[CIT0027] Barrett KA, Nakao JH, Taylor EV, Eggers C, Gould LH. 2017. Fish-associated foodborne disease outbreaks: United States, 1998–2015. Foodborne Pathog Dis. 14(9):537–543. 28682115 10.1089/fpd.2017.2286

[CIT0028] Barros MB, Paes R, Schubach AO. 2011. *Sporothrix schenckii* and Sporotrichosis. Clin Microbiol Rev. 24(4):633–654. 21976602 10.1128/CMR.00007-11 PMC3194828

[CIT0029] Benie CKD, Dadié A, Guessennd N, N’gbesso-Kouadio NA, Kouame ND, N’golo DC, Aka S, Dako E, Dje KM, Dosso M. 2017. Characterization of virulence potential of *Psudomonas aeruginosa* isolated from bovine meat, fresh fish, and smoked fish. Eur J Microbiol Immunol. 7(1):55–64. [CrossRef*]*[10.1556/1886.2016.00039] PMC5372481 28386471

[CIT0030] Berzak R, Scheinin A, Davidovich N, Regev Y, Diga R, Tchernov D, Morick D. 2019. Prevalence of nervous necrosis virus (NNV) and *Streptococcus* species in wild marine fish and crustaceans from the Levantine Basin, Mediterranean Sea. Dis Aquat Organ. 133(1):7–17. 30997880 10.3354/dao03339

[CIT0031] Bhambri S, Bhambri A, Del Rosso JQ. 2009. Atypical mycobacterial cutaneous infections. Dermatol Clin. 27(1):63–73. 18984369 10.1016/j.det.2008.07.009

[CIT0032] Bibi F, Qaisrani SN, Ahmad AN, Akhtar M, Khan BN, Ali A. 2015. Occurrence of *Salmonella* in freshwater fishes: a review. J Anim Plant Sci. 25(3):303–310.

[CIT0033] Bigliazzi C, Poletti V, Dell’Amore D, Saragoni L, Colby TV. 2004. Disseminated basidiobolomycosis in an immunocompetent woman. J Clin Microbiol. 42(3):1367–1369. 15004122 10.1128/JCM.42.3.1367-1369.2004 PMC356830

[CIT0034] Bittencourt AL, Londero AT, Araujo MS, Mendonca N, Bastos JL. 1979. Occurrence of subcutaneous zygomycosis caused by *Basidiobolus haptosporus* in Brazil. Mycopathologia. 68(2):101–104. 573856 10.1007/BF00441089

[CIT0035] Boerlage AS, Graat EAM, Verreth JA, Jong MCM. 2013. Effect of control strategies on the persistence of fish-derived zoonotic trematodes: a modelling approach. Aquaculture. 408-409:106–112.

[CIT0036] Bonifaz A, Sául A, Paredes-Solis V, Fierro L, Rosales A, Palacios C, Araiza J. 2007. Sporotrichosis in childhood: clinical and therapeutic experience in 25 patients. Pediatr Dermatol. 24(4):369–372. 17845157 10.1111/j.1525-1470.2007.00452.x

[CIT0037] Bonyadian M, Fardizad H, Akbarian A, Karimi Ghahfarokh F. 2014. Pool water and Rainbow trout contamination to some enteric bacteria in Chaharmahalva Bakhtiari province. Int J Virt Real. 10(3): 94–99.

[CIT0038] Borges JN, Cunha LFG, Santos HLC, Monteiro-Neto C, Santos CP. 2012. Morphological and molecular diagnosis of anisakid nematode larvae from Cutlassfish (*Trichiurus lepturus*) off the Coast of Rio de Janeiro, Brazil. PLoS One. 7(7):e40447. 22792329 10.1371/journal.pone.0040447 PMC3392247

[CIT0039] Boylan S. 2011. Zoonoses associated with fish. Vet Clin North Am Exot Anim Pract. 14(3):427–438. 21872780 10.1016/j.cvex.2011.05.003

[CIT0040] Bujan N, Toranzo A, Magarinos B. 2018. *Edwardsiella piscicida*: a significant bacterial pathogen of cultured fish. Dis Aqu Org. 131(1): 59–71. 10.3354/dao03281 30324915

[CIT0041] Butt AA, Aldridge KE, Sander CV. 2004. Infections related to the ingestion of seafood. Part II: parasitic infections and food safety. Lancet Infect Dis. 4(5):294–300. 15120346 10.1016/S1473-3099(04)01005-9

[CIT0042] Caffara M, Gustinelli A, Mazzone A, Fioravanti ML. 2020. Multiplex PCR for simultaneous identification of the most common European Opisthorchiid and Heterophyid in fish or fish products. Food Waterborne Parasitol. 19:e00081. 32435707 10.1016/j.fawpar.2020.e00081 PMC7232091

[CIT0043] Camacho SPD, Willms K, de la Cruz MDC, Ramos MLZ, Gaxiola SB, Velázquez RC, Gonzáles SS. 2003. Acute outbreak of gnathostomiasis in a fishing community in Sinaloa, Mexico. Parasitol Int. 52(2):133–140. 12798924 10.1016/s1383-5769(03)00003-5

[CIT0044] Cardozo MV, Borges CA, Beraldo LG, Maluta RP, Pollo AS, Borzi MM, Santos LF, Kariyawasam S, Avila FA. 2018. Shigatoxigenic and atypical enteropathogenic *Escherichia coli* in fish for human consumption. Braz J Microbiol. 49(4):936–941. 29891415 10.1016/j.bjm.2018.02.013 PMC6175746

[CIT0045] Carmona-Salido H, Fouz B, Sanjuán E, Carda M, Delannoy CMJ, García-González N, González-Candelas F, Amaro C. 2021. The widespread presence of a family of fish virulence plasmids in Vibrio vulnificus stresses its relevance as a zoonotic pathogen linked to fish farms. Emerg Microbes Infect. 10(1):2128–2140. 34702148 10.1080/22221751.2021.1999177 PMC8635547

[CIT0046] Carson J, Wilson T, Douglas M, Barnes A. 2019. Yersiniosis in fish. Australian and New Zealand Standard Diagnostic Procedures (ANZSDP) for Yersiniosis in fish. Centre for Aquatic Animal Health and Vaccines Animal Health Laboratory Department of Primary Industries and Water, Launceston, Tasmania, 7250 and The University of Queensland School of Biological Sciences and Centre for Marine Science, St Lucia Campus, Brisbane, Queensland.

[CIT0048] Center of Disease Control and Prevention (CDC). 2016. Norovirus: clinical overview. www.cdc.gov.

[CIT0049] Centers for Disease Control and Prevention (CDC). 2017. Fungal diseases. Centers for Disease Control and Prevention.

[CIT0050] Chai J-Y, Darwin Murrell K, Lymbery AJ. 2005. Fish-borne parasitic zoonoses: status and issues . Int J Parasitol. 35(11-12):1233–1254. 16143336 10.1016/j.ijpara.2005.07.013

[CIT0051] Chai JY, Shin EH, Lee SH, Rim HJ. 2009. Foodborne intestinal flukes in Southeast Asia. Korean J Parasitol. 47(Suppl):S69–S102. 19885337 10.3347/kjp.2009.47.S.S69 PMC2769220

[CIT0052] Chen H. 1995. Seafood microorganisms and seafood safety. J Food Drug Anal. 3:133–144.

[CIT0053] Chinabut S. 1999. Fish disease and disorders: Viral, bacterial, and fungal infections. 2nd ed. (Woo PT, Bruno DW, eds.). Wallingford (UK): CAB International, 3.

[CIT0054] Choi BI, Han JK, Hong ST, Lee KH. 2004. Clonorchiasis and cholangiocarcinoma: etiologic relationship and imaging diagnosis. Clin Microbiol Rev. 17(3):540–552. 15258092 10.1128/CMR.17.3.540-552.2004 PMC452546

[CIT0055] Chowdhury CA, Aleem M, Khan MSI, Hossain ME, Ghosh S, Z, Rahman M. 2021. Major zoonotic diseases of public health importance in Bangladesh. Vet Med Sci. 7(4):1199–1210. 33650812 10.1002/vms3.465 PMC8013274

[CIT0056] Clausen JH, Madsen H, Murrell KD, Van PT, Thu HNT, Do DT, Thi LAN, Manh HN, Dalsgaard A. 2012. Prevention and control of fish-borne zoonotic trematodes in fish nurseries, Vietnam . Emerg Infect Dis. 18(9):1438–1445. 22932069 10.3201/eid1809.111076 PMC3437734

[CIT0057] Collins MD, East AK. 1998. Phylogeny and taxonomy of the food-borne pathogen *Clostridium botulinum* and its neurotoxins. J Appl Microbiol. 84(1):5–17. 15244052 10.1046/j.1365-2672.1997.00313.x

[CIT0058] Cong W, Elsheikha HM. 2021. Biology, epidemiology, clinical features, diagnosis, and treatment of selected fish-derived parasitic zoonoses. Yale J Biol Med. 94(2):297–309. 34211350 PMC8223542

[CIT0059] da Rosa ACM, Scroferneker ML, Vettorato R, Gervini RL, Vettorato G, Weber A. 2005. Epidemiology of sporotrichosis: a study of 304 cases in Brazil. J Am Acad Dermatol. 52(3 Pt 1):451–459. 15761423 10.1016/j.jaad.2004.11.046

[CIT0060] Daengsvang S. 1981. Gnathostomiasis in Southeast Asia. Southeast Asian J Trop Med Public Health. 12(3):319–332. 7342319

[CIT0061] Das A, Acharya S, Behera BK, Paria P, Bhowmick S, Parida PK, Das BK. 2018. Isolation, identification and characterization of *Klebsiella pneumoniae* from infected farmed Indian Major Carp *Labeorohita* (Hamilton 1822) in West Bengal, India. Aquaculture. 482:111–116.

[CIT0062] Davies YM, Oliveira MGX, Cunha MPV, Franco LS, Santos LSP, Moreno LZ, de Moura Gomes VT, Sato MIZ, Nardi MS, Moreno AM, et al. 2018. *Edwardsiella tarda* outbreak affecting fishes and aquatic birds in Brazil. Vet Q. 38(1):99–105. 30668277 10.1080/01652176.2018.1540070 PMC6830998

[CIT0063] Deardorff TL. 1991. Epidemiology of marine fish-derived parasitic zoonoses. Southeast Asian J Trop Med Public Health. 22:146–149. 1822874

[CIT0064] Delghandi MR, El-Matbouli M, Menanteau-Ledouble S. 2020a. Mycobacteriosis and infections with nontuberculous mycobacteria in aquatic organisms: a review. Microorganisms. 8(9):1368. 10.3390/microorganisms8091368 PMC7564596 32906655

[CIT0065] Delghandi MR, Waldner K, El-Matbouli M, Menanteau-Ledouble S. 2020b. Identification Mycobacterium spp. in the natural water of two Austrian rivers. Microorganisms. 8(9):1305. 10.3390/microorganisms8091305 PMC7563569 32867056

[CIT0066] Diana TC, Manjulatha C. 2012. Incidence and identification of *Klebsiella pneumoniae* in mucosal buccal polyp of *Nemipterus japonicus* of Visakhapatnam Coast, India. J Foot Ankle Surg. 7(6):454–460.

[CIT0067] Dick TA. 2007. Diphyllobothriasis: the *Diphyllobothrium latum* human infection conundrum and reconciliation with a worldwide zoonosis. In: Food-borne parasitic zoonoses. Boston (MA): Springer; 11:151–184.

[CIT0068] Dorny P, Praet N, Deckers N, Gabriel S. 2009. Emerging food-borne parasites. Vet Parasitol. 163(3):196–206. 19559535 10.1016/j.vetpar.2009.05.026

[CIT0069] dos Santos CAML, Howgate P. 2011. Fishborne zoonotic parasites and aquaculture: a review. Aquaculture. 318(3-4):253–261.

[CIT0070] Durborow RM. 1999. Health and safety concerns in fisheries and aquaculture. J Foot Ankle Surg. 14(2):373–406. 10329911

[CIT0071] Eiras JC, Pavanelli GC, Takemoto RM, Nawa Y. 2018a. Fish-borne nematodiases in South America: neglected emerging diseases . J Helminthol. 92(6):649–654. 29067898 10.1017/S0022149X17001006

[CIT0072] Eiras JC, Pavanelli GC, Takemoto RM, Nawa Y. 2018b. An overview of fish-borne nematodiases among returned travelers for recent 25 years- unexpected Diseases sometimes far away from the origin . Korean J Parasitol. 56(3):215–227. 29996625 10.3347/kjp.2018.56.3.215 PMC6046559

[CIT0073] Elham MI, Mona MI, Maather ME, Heba IA. 2017. Studies on *Pseudomonas* septicemia in some tilapia in Ismailia. Suez Canal Vet Med J. 22(1):107–117.

[CIT0074] El-Seify MA, Sultan K, Elhawary NM, Satour NS, Marey NM. 2021. Prevalence of heterophyid infection in tilapia fish ‘*Orechromas niloticus’* with emphasize of cats role as neglected reservoir for zoonotic *Heterophyes heterophyes* in Egypt. J Parasit Dis. 45(1):35–42. 33746384 10.1007/s12639-020-01277-7 PMC7921218

[CIT0075] El-Shabrawi MH, Kamal NM. 2011. Gastrointestinal basidiobolomycosis in children: an overlooked emerging infection. J Med Microbiol. 60(Pt 7):871–880. 21546558 10.1099/jmm.0.028670-0

[CIT0076] Enes JE, Wages AJ, Malone JB, Tesana S. 2010. Prevalence of *Opisthorchis viverrini* infection in the canine and feline hosts in three villages, KhonKaen Province, northeastern Thailand. Southeast Asian J Trop Med Public Health. 41(1):36–42. 20578480 PMC3777564

[CIT0077] Environmental Health and Safety (EHS)/Occupational Health. 2016. Zoonotic Diseases–Fish. University of Colorado Denver/Anschutz Medical Campus. https://www.ucdenver.edu/.

[CIT0078] Epps SV, Harvey RB, Hume ME, Phillips TD, Anderson RC, Nisbet DJ. 2013. Foodborne *Campylobacter:* infections, metabolism, pathogenesis and reservoirs. Int J Environ Res Public Health. 10(12):6292–6304. 24287853 10.3390/ijerph10126292 PMC3881114

[CIT0079] Eriksen T, Brantsaeter AB, Kiehl W, Steffens I. 2004. Botulism infection after eating fish in Norway and Germany: two outbreak reports. Weekly Release. 8(3):2366.

[CIT0080] Espelund M, Klaveness D. 2014. Botulism outbreaks in natural environments – an update. Front Microbiol. 5:287. 24966853 10.3389/fmicb.2014.00287 PMC4052663

[CIT0081] Facciola A, Riso R, Avventuroso E, Visalli G, Delia SA, Laganà P. 2017. *Campylobacter*: from microbiology to prevention. J Prev Med Hyg. 58(2):E79–E92. 28900347 PMC5584092

[CIT0082] Faeed M, Mozafari NA, ShojaeeArany A. 2005. Isolation and identification of bacteria and fungi of spoilage in kilka meal production in Gilan province. Iranian Sci Fish J. 4(4):127–138.

[CIT0083] Farzadnia A, Naeemipour M. 2020. Molecular techniques for the detection of bacterial zoonotic pathogens in fish and humans. Aquacult Int. 28(1):309–320.

[CIT0084] Fernandes MR, Sellera FP, Moura Q, Carvalho MPN, Rosato PN, Cerdeira L, Lincopan N. 2018. Zooanthroponotic transmission of drug-resistant *Pseudomonas aeruginosa*, Brazil. Emerg Infect Dis. 24(6):1160–1162. 29774849 10.3201/eid2406.180335 PMC6004847

[CIT0085] Fioravanti ML, Gustinelli A, Rigos G, Buchmann K, Caffara M, Pascual S, Pardo MÁ. 2021. Negligible risk of zoonotic anisakid nematodes in farmed fish from European mariculture, 2016 to 2018. Euro Surveill. 26(2):1900717. 10.2807/1560-7917.ES.2021.26.2.1900717 PMC7809721 33446302

[CIT0086] Fiorenza EA, Wendt CA, Dobkowski KA, King TL, Pappaionou M, Rabinowitz P, Samhouri JF, Wood CL. 2020. It’s a wormy world: Meta-analysis reveals several decades of change in the global abundance of the parasitic nematodes *Anisakis* spp. and *Pseudoterranova* spp. in marine fishes and invertebrates . Glob Chang Biol. 26(5):2854–2866. 32189441 10.1111/gcb.15048

[CIT0087] Fleury RN, Taborda PR, Gupta AK, Fujita MS, Rosa PS, Weckwerth AC, Negrão MS, Bastazini I. 2001. Zoonotic sporotrichosis. Transmission to humans by infected domestic cat scratching: report of four cases in São Paulo, Brazil. Int J Dermatol. 40(5):318–322. 11575308

[CIT0088] Freedman JC, Shrestha A, McClane BA. 2016. *Clostridium perfringens* enterotoxin: action, genetics, and translational applications. Toxins (Basel). 8(3):73. 10.3390/toxins8030073 PMC4810218 26999202

[CIT0089] Friesema I, de Jong A, Hofhuis A, Heck M, van den Kerkhof H, de Jonge R, Hameryck D, Nagel K, van Vilsteren G, van Beek P, et al. 2014. Large outbreak of *Salmonella* Thompson related to smoked salmon in the Netherlands, August to December 2012. Eurosurveillance. 19(39):20918. 25306981 10.2807/1560-7917.es2014.19.39.20918

[CIT0090] Fürst T, Keiser J, Utzinger J. 2012. Global burden of human food-borne trematodiasis: a systematic review and meta-analysis. Lancet Infect Dis. 12(3):210–221. 22108757 10.1016/S1473-3099(11)70294-8

[CIT0091] Gauthier DT. 2015. Bacterial zoonoses of fishes: a review and appraisal of evidence for linkages between fish and human infections. Vet J. 203(1):27–35. 25466575 10.1016/j.tvjl.2014.10.028

[CIT0092] Gcebe N, Michel AL, Hlokwe TM. 2018. Non-tuberculous *Mycobacterium* species causing mycobacteriosis in farmed aquatic animals of South Africa. BMC Microbiol. 18(1):32–11. 29653505 10.1186/s12866-018-1177-9 PMC5899368

[CIT0093] Germann R, Schächtele M, Nessler G, Seitz U, Kniehl E. 2003. Cerebral gnathostomiasis as a cause of an extended intracranial bleeding. Klin Padiatr. 215(4):223–225. 12929012 10.1055/s-2003-41401

[CIT0094] Golomazou E, Malandrakis EE, Panagiotaki P, Karanis P. 2021. *Cryptosporidium* in fish: implications for aquaculture and beyond. Water Res. 201:117357. 34147739 10.1016/j.watres.2021.117357

[CIT0095] Gopi M, Thankappanpillai T, Kumar A, Prakash S. 2016. Opportunistic pathogen *Klebsiella pneumoniae* isolated from Maldive’s clown fish *Amphiprion nigripes* with hemorrhages at Agatti Island, Lakshadweep archipelago. Int J Fish Aquat Studies. 4(3):464–467.

[CIT0096] Grant S, Olsen C. 1999. Preventing zoonotic diseases in immunocompromised persons: the role of physicians and veterinarians. Emerg Infect Dis. 5(1):159–163. 10081686 10.3201/eid0501.990121 PMC2627689

[CIT0097] Gugnani HC. 1999. A review of zygomycosis due to *Basidiobolus ranarum*. Eur J Epidemiol. 15(10):923–929. 10669127 10.1023/a:1007656818038

[CIT0098] Guillen G, Wrast J. 2010. Fishes as sources of E. coli bacteria in warm water streams, Final Report of Project managed by the Texas Water Resources Institute. p. 81.

[CIT0099] Gundogan N. 2014. Occurrence of *Klebsiella* in humans, foods, waters, and environments. In: Batt CA, Tortorello ML, editors. ScienceDirect, Encyclopedia of food microbiology, 2nd ed.

[CIT0100] Guzman E, Shotts EB, Gratzek JB. 2013. Review of bacterial diseases of aquarium fish. International Association for Aquatic Animal Medicine (IAAAM) Conference. 1986.

[CIT0101] Haddad V, Miot HA, Bartoli LD, Cardoso ADC, De Camargo RMP. 2002. Localized lymphatic sporotrichosis after fish-induced injury (*Tilapia* spp.). Med Mycology. 40(4):425–427. 10.1080/mmy.40.4.425.427 12230224

[CIT0102] Haile AB, Getahun TK. 2018. Isolation and identification of *Escherichia coli* and *Edwardsiellatarda* from fish harvested for human consumption from Zeway Lake, Ethiopia. African J Microbiol Res. 12(20):476–480.

[CIT0103] Han BA, Kramer AM, Drake JM. 2016. Global patterns of zoonotic disease in mammals. Trends Parasitol. 32(7):565–577. 27316904 10.1016/j.pt.2016.04.007 PMC4921293

[CIT0104] Hansen DL, Clark JJ, Ishii S, Sadowsky MJ, Hicks RE. 2008. Sources and sinks of *Escherichia coli* in Benthic and Pelagic fish. J Great Lakes Res. 34(2):228–234.2.0.CO;2][Mismatch

[CIT1233] Haenen OLM, Evans JJ, Berthe F. 2013. Bacterial Infections From Aquatic Species: Potential For and Prevention Of Contact Zoonoses. Rev Sci Tech Off Epiz. 32(2):497–507. 10.20506/rst.32.2.2245 24547653

[CIT0105] Hashish E, Merwad AMA, ElgamiSh A, Amer A, Kamal H, Elsadek A, Marei A, Sitohy M. 2018. *Mycobacterium marinum* infection in fish and man: epidemiology, pathophysiology and management; a review. Vet Q. 38(1):01–34. 10.1080/01652176.2018.1447171 PMC6831007 29493404

[CIT4233] Hedegaard Clausen J, Madsen H, Murrell KD, Van PT, Thu HN, Do DT, Nguyen Thi LA, Nguyen Manh H, Dalsgaard A. 2012. Prevention and Control Of Fish-Borne Zoonotic Trematodes In Fish Nurseries, Vietnam. Emerg Infect Dis. 18(9):1438–45. 10.3201/eid1809.111076. PMC3437734 22932069

[CIT0106] Helmi M, Mukti AT, Soegianto A, Effendi MH. 2020. A review of vibriosis in fisheries: public health importance. Sys Rev Pharm. 11(8):51–58.

[CIT0107] Herman JS, Chiodini PL. 2009. Gnathostomiasis, another emerging imported disease. Clin Microbiol Rev. 22(3):484–492. 19597010 10.1128/CMR.00003-09 PMC2708391

[CIT0108] Hernández-Cabanyero C, Amaro C. 2020. Phylogeny and life cycle of the zoonotic pathogen *Vibrio vulnificus*. Environ Microbiol. 22(10):4133–4148. 32567215 10.1111/1462-2920.15137

[CIT0109] Hossen MS, Wassens S, Shamsi S. 2021. Occurrence and abundance of zoonotic nematodes in snapper *Chrysophrys auratus*, a popular table fish from Australian and New Zealand waters. Food Waterborne Parasitol. 23:e00120. 33817358 10.1016/j.fawpar.2021.e00120 PMC8010209

[CIT0110] Hung NM, Dung DT, Anh NTL, Van PT, Thanh BN, Ha NV, Hien HV, Canh LX. 2015. Current status of fish-derived zoonotic trematode infections in Gia Vien district, NinhBinh province, Vietnam. Parasit Vectors. 8(1):21. 25586313 10.1186/s13071-015-0643-6 PMC4299372

[CIT0111] Huss HH, Reilly A, Ben Embarek PK. 2000. Prevention and control of hazards in seafood. Food Control. 11(2):149–156.

[CIT0112] Huzmi H, Ina-Salwany MY, Natrah FMI, Syukri F, Karim M. 2019. Strategies of controlling vibriosis in fish. Asian-Australasian J Anim Sci. 7(5):513–521.

[CIT0113] Iregui CA, Comas J, Vasquez GMV, Verjan N. 2016. Experimental early pathogenesis of *Streptococcus agalactiae* infection in red tilapia *Oreochromis* spp. J Fish Dis. 39(2):205–215. 25683349 10.1111/jfd.12347

[CIT0114] Itoh M, Okamoto S, Kariya H. 1986. Survey of 200 cases of sporotrichosis. Dermatologica. 172(4):209–213. 3709907 10.1159/000249337

[CIT0115] Jami M, Ghanbar M, Zunabovic M, Domig KJ, Kneifel W. 2014. *Listeria monocytogenes* in aquatic food products-A review. Comp Rev Food Sci Food Safety. 13(5):798–813.

[CIT0116] Jin L, Chen Y, Yang W, Qiao Z, Zhang X. 2020. Complete genome sequence of fish-pathogenic Aeromonas hydrophila HX-3 and a comparative analysis: insights into virulence factors and quorum sensing . Sci Rep. 10(1):15479. 32968153 10.1038/s41598-020-72484-8 PMC7512022

[CIT0117] Jones SRM. 2015. Transmission dynamics of foodborne parasites in fish and shellfish. In: Foodborne parasites in the food supply web occurrence and control. Woodhead Publishing: Woodhead Publishing Series in Food Science, Technology and Nutrition, 293–315.

[CIT0118] Jones KE, Patel NG, Levy MA, Storeygard A, Balk D, Gittleman JL, Daszak P. 2008. Global trends in emerging infectious diseases. Nature. 451(7181):990–993. 18288193 10.1038/nature06536 PMC5960580

[CIT0119] Jun L, Woo YS. 2003. Pathogenicity of *Vibrio* in fish. J Ocean Univ China. 2(2):117–128.

[CIT0120] Kalimuddin S, Chen SL, Lim CTK, Koh TH, Tan TY, Kam M, Wong CW, Mehershahi KS, Chau ML, Ng LC, et al. 2017. 2015 epidemic of severe *Streptococcus agalactiae* sequence type 283 infections in Singapore associated with the consumption of raw freshwater fish: a detailed analysis of clinical, epidemiological, and bacterial sequencing data. Clin Infect Dis. 64(suppl_2):S145–S152. 28475781 10.1093/cid/cix021

[CIT0121] Karsidani SH, Soltani M, Nikbakhat-Brojeni G, Ghasemi M, Skall H. 2010. Molecular epidemiology of zoonotic streptococcosis/lactococcosis in rainbow trout (*Oncorhynchus mykiss*) aquaculture in Iran. Iran J Microbiol. 2(4):198–209. 22347573 PMC3279792

[CIT0122] Kenyon EM, Russell LH, McMurray DN. 1984. Isolation of *Sporothrix schenckii* from potting soil. Mycopathologia. 87(1-2):128. 6493313 10.1007/BF00436641

[CIT0123] Kerie Y, Nuru A, Abayneh T. 2019. *Edwardsiella*species infection in fish population and its status in Ethiopia. J Fish Aquacult. 10:2.

[CIT0124] Keukeleire SD, Bel AD, Jansen Y, Janssens M, Wauters G, Pierard D. 2014. *Yersinia ruckeri*, an unusual microorganism isolated from a human wound infection. New Microbes New Infect. 2(4):134–135. 25356360 10.1002/nmi2.56 PMC4184584

[CIT0125] Khan ZU, Khoursheed M, Makar R, Al-Waheeb S, Al-Bader I, Al-Muzaini A, Makar R, Al-Waheeb S, Al-Bader I, Al-Muzaini A, et al. 2001. *Basidiobolus ranarum* as an etiologic agent of gastrointestinal zygomycosis. J Clin Microbiol. 39(6):2360–2363. 11376094 10.1128/JCM.39.6.2360-2363.2001 PMC88148

[CIT0126] Khardori N, Fainstein V. 1988. *Aeromonas* and *Plesiomonas* as etiological agents. Annu Rev Microbiol. 42:395–419. 3059998 10.1146/annurev.mi.42.100188.002143

[CIT0127] Kittigul L, Thamjaroen A, Chiawchan S, Chavalitshewinkoon-Petmitr P, Pombubpa K, Diraphat P. 2016. Prevalence and molecular genotyping of Noroviruses in market oysters, mussels, and cockles in Bangkok, Thailand. Food Environ Virol. 8(2):133–140. 26872638 10.1007/s12560-016-9228-6

[CIT0128] Kumar G, Menanteau-Ledouble S, Saleh M, El-Matbouli M. 2015. *Yersinia ruckeri*, the causative agent of enteric redmouth disease in fish. Vet Res. 46:103. 26404907 10.1186/s13567-015-0238-4 PMC4581093

[CIT0129] Kwon-Chung KJ, Bennett JE. 1992. Medical mycology. 2nd ed., Philadelphia: Lea and Febiger; p. 469–512.

[CIT0130] Lassen SJ, Ethelberg S, Bjorkman JT, Jensen T, Sørensen G, Kvistholm Jensen A, Müller L, Nielsen EM, Mølbak K. 2016. Two *Listeria* outbreaks caused by smoked fish consumption-using whole-genome sequencing for outbreak investigations . Clin Microbiol Infect. 22(7):620–624. 27145209 10.1016/j.cmi.2016.04.017

[CIT0131] Leal CAG, Queiroz GA, Pereira FL, Tavares GC, Figueiredo HCP. 2019. *Streptococcus agalactiae* sequence type 283 in farmed fish, Brazil. Emerg Infect Dis. 25(4):776–779. 30882311 10.3201/eid2504.180543 PMC6433023

[CIT0132] Lehane L, Rawlin GT. 2000. Topically acquired bacterial zoonoses from fish: a review. Med J Aust. 173(5):256–259. 11130351 10.5694/j.1326-5377.2000.tb125632.x

[CIT0133] Lehel J, Yaucat-Guendi R, Darnay L, Palotás P, Laczay P. 2021. Possible food safety hazards of ready-to-eat raw fish containing product (Sushi, Sashimi). Crit Rev Food Sci Nutr. 61(5):867–888. Epub 2020 Apr 9. PMID: 32270692. 32270692 10.1080/10408398.2020.1749024

[CIT0134] Leung KY, Wang Q, Yang Z, Siame BA. 2019. *Edwardsiella piscicida*: a versatile emerging pathogen of fish. Virulence. 10(1):555–567. 31122125 10.1080/21505594.2019.1621648 PMC6592360

[CIT0135] Li D, Stals A, Tang QJ, Uyttendaele M. 2014. Detection of noroviruses in shellfish and semiprocessed fishery products from a Belgian seafood company. J Food Prot. 77(8):1342–1347. 25198595 10.4315/0362-028X.JFP-14-016

[CIT0136] Lin R, Li X, Lan C, Yu S, Kawanaka M. 2005. Investigation on the epidemiological factors of *Clonorchis sinensis* infection in an area of south China. Southeast Asian J Trop Med Public Health. 36(5):1114–1117. 16438134

[CIT0137] Liu GH, Sun MM, Elsheikha HM, Fu YT, Sugiyama H, Ando K, Sohn WM, Zhu XQ, Yao C. 2020. Human gnathostomiasis: a neglected food-borne zoonosis. Parasit Vectors. 13(1):616. 33298141 10.1186/s13071-020-04494-4 PMC7724840

[CIT0138] Lohmus M, Bjorklund M. 2015. Climate change: what will it do to fish—parasite interactions? Biol J Linn Soc. 116(2):397–411.

[CIT0139] Lowry T, Smith SA. 2007. Aquatic zoonoses associated with food, bait, ornamental, and tropical fish. J Am Vet Med Assoc. 231(6):876–880. 17867970 10.2460/javma.231.6.876

[CIT0140] Mahajan VK. 2014. Sporotrichosis: an overview and therapeutic options. Dermatol Res Pract. 2014:272376. 25614735 10.1155/2014/272376 PMC4295339

[CIT0141] Mahajan VK, Sharma NL, Sharma RC, Gupta ML, Garg G, Kanga AK. 2005. Cutaneous sporotrichosis in Himachal Pradesh, India. Mycoses. 48(1):25–31. 15679662 10.1111/j.1439-0507.2004.01058.x

[CIT0142] Maleewong W, Wongkham C, Intapan P, Pariyanonda S, Morakote N. 1992. Excretory-secretory antigenic components of *Paragonimus heterotremus* recognized by infected human sera. J Clin Microbiol. 30(8):2077–2079. 1500515 10.1128/jcm.30.8.2077-2079.1992 PMC265445

[CIT0143] Manivong K, Komalamisra C, Waikagul J, Radomyos P. 2009. *Opisthorchis viverrini* metacercariae in cyprinoid Fish from three rivers in Khammouane province, Lao PDR. J Trop Med Parasitol. 32:23–29.

[CIT0144] Mantadakis E, Samonis G. 2009. Clinical presentation of zygomycosis. Clin Microbiol Infect. 15(5):15–20. 10.1111/j.1469-0691.2009.02974.x 19754751

[CIT0145] Marbán-Castro E, Mattar S, González Tous M. 2019. Reemerging zoonoses under the ‘One Health’ approach. Magazine MVZ Córdoba. 24(3):7280–7284. [Mismatch

[CIT0146] Marcogliese DJ. 2003. Food webs and biodiversity: are parasites the missing link. J Parasitol. 89(6):106–113.

[CIT0147] Marsh Z, Shah MP, Wikswo ME, Barclay L, Kisselburgh H, Kambhampati A, Cannon JL, Parashar UD, Vinjé J, Hall AJ. 2018. Epidemiology of foodborne Norovirus outbreaks - United States, 2009-2015. Food Saf (Tokyo). 6(2):58–66. 32231948 10.14252/foodsafetyfscj.2017028 PMC6989197

[CIT0148] Mayorga R, Cáceres A, Toriello C, Gutiérrez G, Alvarez O, Ramirez ME, Mariat F. 1978. An endemic area of sporotrichosis in Guatemala. Sabouraudia. 16(3):185–198. 360441

[CIT0149] McConnaughey M. 2014. Life cycle of Parasites. In: Biomedical science. Amsterdam, The Netherlands: Elsevier. p. 1–15. 10.1016/B978-0-12-801238-3.05115-1.

[CIT0150] Mendiratta V, Karmakar S, Jain A, Jabeen M. 2012. Severe cutaneous zygomycosis due to *Basidiobolus ranarum* in a young infant. Pediatr Dermatol. 29(1):121–123. 21906146 10.1111/j.1525-1470.2011.01476.x

[CIT0151] Meron D, Davidovich N, Ofek-Lalzar M, Berzak R, Scheinin A, Regev Y, Diga R, Tchernov D, Morick D. 2020. Specific pathogens and microbial abundance within liver and kidney tissues of wild marine fish from the Eastern Mediterranean Sea. Microb Biotechnol. 13(3):770–780. 32059079 10.1111/1751-7915.13537 PMC7111072

[CIT0152] Meurens F, Dunoyer C, Fourichon C, Gerdts V, Haddad N, Kortekaas J, Lewandowska M, Monchatre-Leroy E, Summerfield A, WichgersSchreur PJ, et al. 2021. Animal board invited review: risks of zoonotic disease emergence at the interface of wildlife and livestock systems. Animal. 15(6):100241. 34091225 10.1016/j.animal.2021.100241 PMC8172357

[CIT0153] Migaki G, Font RL, Kaplan W, Asper ED. 1978. Sporotrichosis in a Pacific white-sided dolphin (*Lagenorhynchus obliquidens*). Am J Vet Res. 39:1916–1919. 749575

[CIT0154] Mugerwa JW. 1976. *Subcutaneous phycomycosis* in Uganda. Br J Dermatol. 94(5):539–544. 1268064 10.1111/j.1365-2133.1976.tb05143.x

[CIT0155] Murrell K. 2002. Fish borne zoonotic parasites: epidemiology, detection, and elimination. In: Safety and quality issues in fish processing. Cambridge: Woodhead Publishing Ltd, p. 114–141.

[CIT0156] Murrell KD, Fried B (Eds). 2007. Food-borne parasitic zoonoses. In: Fish and plant-borne parasites. 1st ed. New York, Springer; pp. 429.

[CIT0157] Nakajima H, Inoue M, Mori T. 1991. Isolation of *Yersinia*, *Campylobacter*, *Plesiomonas* and *Aeromonas* from environmental water and fresh water fishes. Nippon KoshuEiseiZasshi. 38:815–820. 1747520

[CIT0158] Nawa Y, Nakamura-Uchiyama F. 2004. An overview of gnathostomiasis in the world. Southeast Asian J Trop Med Public Health. 35(1):87–91.

[CIT0159] Nguyen TH, Dorny P, Nguyen TTG, Dermauw V. 2021. Helminth infections in fish in Vietnam: a systematic review. Int J Parasitol Parasites Wildl. 14:13–32. 33384920 10.1016/j.ijppaw.2020.12.001 PMC7770511

[CIT0160] Nielsen JJ, Blomberg B, Gaïni S, Lundemoen K. 2018. Aortic valve endocarditis with *Erysipelothrix rhusiopathiae*: A rare zoonosis. Infect Dis Rep. 10(3):7770. 30542523 10.4081/idr.2018.7770 PMC6240838

[CIT0161] Nieuwenhuizen NE. 2016. Anisakis - immunology of a foodborne parasitosis. Parasite Immunol. 38(9):548–557. 27428817 10.1111/pim.12349

[CIT0162] Noga E. 2010. Diagnoses made by bacterial culture of kidney or affected organs. In: Fish disease, diagnosis and treatment. 2nd ed. Ames (IA): Iowa State University Press. p. 185–190.

[CIT0163] Novotny L, Dovorska L, Lorencova A, Beran V, Pavik I. 2004. Fish: a potential source of bacterial pathogens for human beings. Vet Med J. 49(9):343–358.

[CIT0164] Obaidat MM, Salman AEB, Lafi SQ. 2015. Prevalence of *Staphylococcus aureus* in imported fish and correlations between antibiotic resistance and enterotoxigenicity. J Food Prot. 78(11):1999–2005. 26555523 10.4315/0362-028X.JFP-15-104

[CIT0165] Odeyemi OA, Ahmad A. 2017. Antibiotic resistance profiling and phenotyping of *Aeromonas* species isolated from aquatic sources. Saudi J Biol Sci. 24(1):65–70. 28053573 10.1016/j.sjbs.2015.09.016 PMC5198916

[CIT0166] Ogbeibu AE, Okaka CE, Oribhabor BJ. 2014. Gastrointestinal helminth parasites community of fish Species in a Niger Delta Tidal Creek, Nigeria. J Ecosys. 2014246283::1–10. P.

[CIT0167] Oh WT, Jun JW, Giri SS, Yun S, Kim HJ, Kim SG, Kim SW, Han SJ, Kwon J, Park SC. 2019. *Staphylococcus xylosus* infection in Rainbow trout (*Oncorhynchus mykiss*) as a primary pathogenic cause of eye protrusion and mortality. Microorganisms. 7(9):330. 10.3390/microorganisms7090330 PMC6780347 31500280

[CIT0168] Okafor JI, Testrake D, Mushinsky HR, Yangco BG. 1984. A *Basidiobolus* sp. and its association with reptiles and amphibians in southern Florida. Sabouraudia. 22(1):47–51. 6538344 10.1080/00362178485380081

[CIT0169] Oliveira RV, Peixoto PG, Ribeiro DS, Araujo MC, do Santos CTB, Hayashi C, Pedreira M, Pelli A. 2014. *Klebsiella pneumoniae* as a main cause of infection in Nishikigoi*Cyprinus carpio* (Carp) by inadequate handling. Braz J Vet Pathol. 7(2):86–88.

[CIT0170] Oliviera RV, Oliviera MC, Pelli A. 2017. Disease infection by Enterobacteriaceae family in fishes: a review. J Microbiol Exp. 4(5):00128.

[CIT0171] Pardo González MÁ, Cavazza G, Gustinelli A, Caffara M, Fioravanti M. 2020. Absence of anisakis nematodes in smoked farmed Atlantic salmon (*Salmo salar*) products on sale in European countries. Ital J Food Saf. 9(4):8615. 33532370 10.4081/ijfs.2020.8615 PMC7844585

[CIT0172] Park SB, Aoki T, Jung TS. 2012. Pathogenesis of and strategies for preventing *Edwardsiella tarda* infection in fish. Vet Res. 43:67. 23035843 10.1186/1297-9716-43-67 PMC3479428

[CIT0173] Pavoni E, Consoli M, Suffredini E, Arcangeli G, Serracca L, Battistini R, Rossini I, Croci L, Losio MN. 2013. Noroviruses in seafood: a 9-year monitoring in Italy. Foodborne Pathog Dis. 10(6):533–539. 23638849 10.1089/fpd.2012.1399

[CIT0174] Phillips Savage ACN, Blake L, Suepaul R, McHugh O, Rodgers R, Thomas C, Oura C, Soto E. 2022. Piscine mycobacteriosis in the ornamental fish trade in Trinidad and Tobago. J Fish Dis. Jan 9. Epub ahead of print. PMID: 35000204. 10.1111/jfd.13580. 35000204

[CIT0175] Pinheiro RH, Santana RLS, Melo FTV, Santos JN, Giese EG. 2017. Gnathostomatidae nematode parasite of *Colomesus psittacus* (Osteichthyes, Tetraodontiformes) in the Ilha de Marajó, Brazilian Amazon. Rev Bras Parasitol Vet. 26(3):340–347. 28977248 10.1590/S1984-29612017047

[CIT0176] Polley L, Thompson RC. 2009. Parasite zoonoses and climate change: molecular tools for tracking shifting boundaries. Trends Parasitol. 25(6):285–291. 19428303 10.1016/j.pt.2009.03.007

[CIT0177] Pomaranski EK, Griffin MJ, Camus AC, Armwood AR, Shelley J, Waldbieser GC, LaFrentz BR, García JC, Yanong R, Soto E. 2020. Description of *Erysipelothrix piscisicarius* sp. nov., an emergent fish pathogen, and assessment of virulence using a tiger barb (*Puntigrus tetrazona*) infection model. Int J Syst EvolMicrobiol. 70(2):857–867. 10.1099/ijsem.0.003838 31682217

[CIT0178] Pradeep PJ, Suebsing R, Sirthammajak S, Kampeera J, Jitrakorn S, Saksmerprome V, Turner W, Palang I, Vanichviriyakit R, Senapin S, et al. 2016. Evidence of vertical transmission and tissue tropism of streptococcosis from naturally infected red tilapia (*Oreochromis* spp.). Aquacult Rep. 3:58–66.

[CIT0179] Prueksapanich P, Piyachaturawat P, Aumpansub P, Ridtitid W, Chaiteerakij R, Rerknimitr R. 2018. Liver fluke-associated biliary tract cancer. Gut Liver. 12(3):236–245. 28783896 10.5009/gnl17102 PMC5945254

[CIT0180] Puk K, Guz L. 2020. Occurrence of *Mycobacterium* spp. in ornamental fish. Ann Agric Environ Med. 27(4):535–539. 33356057 10.26444/aaem/114913

[CIT0181] Rabie ME, El Hakeem I, Al-Shraim M, Al Skini MS, Jamil S. 2011. Basidiobolomycosi*s* of the colon masquerading as stenotic colon cancer. Case Rep Surg. 2011(1):685460. 22606589 10.1155/2011/685460 PMC3350237

[CIT0182] Rahman MT, Sobur MA, Islam MS, Ievy S, Hossain MJ, El Zowalaty ME, Rahman AT, Ashour HM. 2020. Zoonotic diseases: etiology, impact, and control. Microorganisms. 8(9):1405. 10.3390/microorganisms8091405 PMC7563794 32932606

[CIT0183] Rahmati AR, Kiani B, Afshari A, Moghaddas E, Williams M, Shamsi S. 2020. World-wide prevalence of *Anisakis* larvae in fish and its relationship to human allergic anisakiasis: a systematic review. Parasitol Res. 119(11):3585–3594. 33025215 10.1007/s00436-020-06892-0

[CIT0184] Raissy M. 2017. Bacterial zoonotic disease from fish: a review. J Food Microbiol. 4(2):15–27.

[CIT2233] Ramanan P, Blumberg AK, Mathison B, Pritt BS. 2013. Parametrial Anisakidosis. J Clin Microbiol. 51(10):3430–3434. 10.1128/JCM.01398-13. PMC3811649 23863565

[CIT0185] Ramos P. 2020. Parasites in fishery products – Laboratorial and educational strategies to control. Exp Parasitol. 211:107865. 32101764 10.1016/j.exppara.2020.107865

[CIT0186] Rasetti-Escargueil C, Lemichez E, Popoff M. 2019. Public health risk associated with Botulism as foodborne zoonoses. Toxins. 12(1):17. 10.3390/toxins12010017 PMC7020394 31905908

[CIT0187] Regev Y, Davidovich N, Berzak R, Lau SCK, Scheinin AP, Tchernov D, Morick D. 2020. Molecular identification and characterization of *Vibrio* species and *Mycobacterium* species in wild and cultured marine fish from the Eastern Mediterranean Sea. Microorganisms. 8(6):863. 10.3390/microorganisms8060863 PMC7356242 32517374

[CIT0188] Rippon JW. 1988. Sporotrichosis. In: Rippon JW, editor. Medical mycology–the pathogenic fungi and the pathogenic actinomycetes. 3rd ed. Philadelphia (PA): W. B. Saunders Company, p. 325–352.

[CIT0189] Rukkawattanakul T, Sookrung N, Seesuay W, Onlamoon N, Diraphat P, Chaicumpa W, Indrawattana N. 2017. Human scFvs that counteract bioactivities of *Staphylococcus aureus* TSST-1. Toxins (Basel). 9(2):50. 10.3390/toxins9020050 PMC5331430 28218671

[CIT0190] Sabry M, Abd El-Moein K, Hamza E, Abdel Kader F. 2016. Occurrence of *Clostridium perfringens* types A, E, and C in fresh fish and its public health significance. J Food Prot. 79(6):994–1000. 27296604 10.4315/0362-028X.JFP-15-569

[CIT0191] Sackey A, Ghartey N, Gyasi R. 2017. Subcutaneous basidiobolomycosis: a case report. Ghana Med J. 51(1):43–46. 28959073 10.4314/gmj.v51i1.9 PMC5611946

[CIT0192] Safonova AE, Voronova AN, Vainutis KS. 2021. First report on molecular identification of *Anisakis simplex* in *Oncorhynchus nerka* from the fish market, with taxonomical issues within Anisakidae. J Nematol. 53:e2021-2023. 10.21307/jofnem-2021-023 PMC8039977 33860240

[CIT0193] Saijuntha W, Sithithaworn P, Petney TN, Andrews RH. 2021. Foodborne zoonotic parasites of the family *Opisthorchiidae*. Res Vet Sci. 135:404–411. 33158552 10.1016/j.rvsc.2020.10.024

[CIT0194] Salikin NH, Nappi J, Majzoub ME, Egan S. 2020. Combating parasitic nematode infections, newly discovered antinematode compounds from marine epiphytic bacteria. Microorganisms. 8(12):1963. 1–19. 10.3390/microorganisms8121963 PMC7764037 33322253

[CIT0195] Saravanakumar PS, Eslami P, Zar FA. 1996. *Lymphocutaneous sporotrichosis* associated with a squirrel bite: case report and review. Clin Infect Dis. 23(3):647–648. 8879801 10.1093/clinids/23.3.647

[CIT0196] Sawadpanich K, Chansuk N, Boonroumkaew P, Sadaow L, Rodpai R, Sanpool O, Janwan P, Intapan PM, Maleewong W. 2021. An unusual case of gastric gnathostomiasis caused by *Gnathostoma spinigerum* confirmed by video gastroscopy and morphological and molecular identification. Am J Trop Med Hyg. 104(6):2050–2054. 33901007 10.4269/ajtmh.21-0015 PMC8176491

[CIT0197] Scholz T, Kuchta R. 2016. Fish-derived, zoonotic cestodes (*Diphyllobothrium* and relatives) in cold climates: a never-ending story of neglected and (re)-emergent parasites. Food Waterborne Parasitol. 4:23–38.

[CIT0198] Seafood Health Facts (SHF). 2020. Making smart choices balancing the benefits and risks of seafood consumption resources for healthcare providers and consumers. https://www.seafoodhealthfacts.org/.

[CIT0199] Shamsi S. 2014. Recent advances in our knowledge of Australian anisakid nematodes. Int J Parasitol Parasites Wildl. 3(2):178–187. 25180162 10.1016/j.ijppaw.2014.04.001 PMC4145145

[CIT0200] Shamsi S. 2016. Seafood-borne parasitic diseases in Australia: how much do we know about them? Microbiol Aust. 37(1):27–29.

[CIT0201] Shamsi S. 2019. Seafood-borne parasitic diseases: a “One-Health” approach is meeded. Fishes. 4(1):9.

[CIT0202] Shamsi S. 2020. Seafood-borne parasites in Australia: human health risks, fact or fiction? Microbiol Aust. 41(1):33–37.

[CIT0203] Shamsi S, Butcher AR. 2011. First report of human anisakidosis in Australia. Med J Aust. 194(4):199–200. 21401462 10.5694/j.1326-5377.2011.tb03772.x

[CIT0204] Shamsi S, Sheorey H. 2018. Seafood-borne parasitic diseases in Australia: are they rare or underdiagnosed? Intern Med J. 48(5):591–596. 29722196 10.1111/imj.13786

[CIT0205] Shamsi S, Steller E, Zhu X. 2021. The occurrence and clinical importance of infectious stage of *Echinocephalus* (Nematoda: Gnathostomidae) larvae in selected Australian edible fish. Parasitol Int. 83:102333. 33731302 10.1016/j.parint.2021.102333

[CIT0206] Shamsi S, Suthar J. 2016. A revised method of examining fish for infection with zoonotic nematode larvae. Int J Food Microbiol. 227:13–16. 27043384 10.1016/j.ijfoodmicro.2016.03.023

[CIT0207] Sharma NL, Sharma RC, Gupta ML, Singh P, Gupta N. 1990. Sporotrichosis: study of 22 cases from himachal Pradesh. Indian J Dermatol VenereolLeprol. 56(4):296–298.

[CIT5233] Shimamura Y, Muwanwella N, Chandran S, Kandel G, Marcon N. 2016. Common Symptoms From an Uncommon Infection: Gastrointestinal Anisakiasis. Can J Gastroenterol Hepatol. 5176502. 10.1155/2016/5176502. PMC5075291 27800471

[CIT0208] Shin B, Park W. 2018. Zoonotic diseases and phytochemical medicines for microbial infections in veterinary science: current state and future perspective. Front Vet Sci. 5(166):166–169. 30140679 10.3389/fvets.2018.00166 PMC6095004

[CIT0209] Shreef K, Saleem M, Saeedd MA, Eissa M. 2017. Gastrointestinal basidiobolomycosis: an emerging and a confusing disease in children (a multicenter experience). Eur J Pediatr Surg. 28(2):194–199. 28166589 10.1055/s-0037-1598104

[CIT0210] Singh R, Xess I, Ramavat AS, Arora R. 2008. Basidiobolomycosis: a rare case report. Indian J Med Microbiol. 26(3):265–267. 18695330

[CIT0211] Skowron K, Wiktorczyk N, Grudlewska K, Wałecka-Zacharska E, Paluszak Z, Kruszewski S, Gospodarek-Komkowska E. 2019. Phenotypic and genotypic evaluation of *Listeria monocytogenes* strains isolated from fish and fish processing plants. Ann Microbiol. 69(5):469–482.

[CIT0212] Smith JW, Wootten R. 1978. *Anisakis* and anisakiasis. Adv Parasitol. 16:93–163. 364959 10.1016/s0065-308x(08)60573-4

[CIT0213] Smith SA. 2011. Working with fish, limiting zoonotic diseases. Global Aquaculture Advocate. www.aquaculturealliance.org.

[CIT0214] Sohn WM, Na BK, Cho SH, Ju JW, Kim CH, Hwang MA, No KW, Park JH. 2021. Prevalence and infection intensity of zoonotic trematode metacercariae in fish from Soyang-cheon (stream), in Wanju-gun, Jeollabuk-do, Korea. Korean J Parasitol. 59(3):265–271. 34218598 10.3347/kjp.2021.59.3.265 PMC8255493

[CIT0215] Sripa B, Bethony JM, Sithithaworn P, Kaewkes S, Mairiang E, Loukas A, Mulvenna J, Laha T, Hotez PJ, Brindley PJ. 2011. Opisthorchiasis and *Opisthorchis*-associated cholangiocarcinoma in Thailand and Laos. Acta Trop. 120(Suppl 1):S158–S168. 20655862 10.1016/j.actatropica.2010.07.006 PMC3010517

[CIT0216] Steffen R, deBernardis C, Baños A. 2003. Travel epidemiology – a global perspective. Int J Antimicrob Agents. 21(2):89–95. 12615369 10.1016/s0924-8579(02)00293-5

[CIT0217] Suthar J, Shamsi S. 2021. The occurrence and abundance of infective stages of zoonotic nematodes in selected edible fish sold in Australian fish markets. Microb Pathog. 154:104833. 33711427 10.1016/j.micpath.2021.104833

[CIT0218] Tantrawatpan C, Intapan PM, Janwan P, Sanpool O, Lulitanond V, Srichantaratsamee C, Anamnart W, Maleewong W. 2013. Molecular identification of *Paragonimus* species by DNA pyrosequencing technology. Parasitol Int. 62(3):341–345. 23246361 10.1016/j.parint.2012.11.008

[CIT0219] Tobback E, Decostere A, Hermans K, Haesebrouck F, Chiers K. 2007. *Yersinia ruckeri* infections in salmonid fish. J Fish Dis. 30(5):257–268. 17501736 10.1111/j.1365-2761.2007.00816.x

[CIT0220] Toranzo AE, Magarinos B, Romalde JL. 2005. A review of the main bacterial fish diseases in mariculture systems. Agriculture. 246(1-4):37–61.

[CIT0221] Tran AKT, Doan HT, Do AN, Nguyen VT, Hoang SX, Le HTT, Hoang HT, Le NH, Le QBT, Le TA. 2019. Prevalence, species distribution, and related factors of fish-derived trematode infection in NinhBinh Province, Vietnam. Biomed Res Int. 30:8581379. 10.1155/2019/8581379 PMC6699318 31467915

[CIT0222] Traoré O, Nyholm o, Siitonen A, Bonkoungou IJO, Traoré AS, Barro N, Haukka K. 2015. Prevalence and diversity of *Salmonella enterica* in water, fish and lettuce in Ouagadougou, Burkina Faso. BMC Microbiol. 15:151. 26228572 10.1186/s12866-015-0484-7 PMC4521495

[CIT0224] Uzal FA, Freedman JC, Shrestha A, Theoret JR, Garcia J, Awad MM, Adams V, Moore RJ, Rood JI, McClane BA. 2014. Towards an understanding of the role of *Clostridium perfringens* toxins in human and animal disease. Future Microbiol. 9(3):361–377. 24762309 10.2217/fmb.13.168 PMC4155746

[CIT0225] Vaiyapuri M, Joseph T, Rao BM, Lalitha KV, Prasad MM. 2019. Methicillin-resistant *Staphylococcus aureus* in seafood: prevalence, laboratory detection, clonal nature, and control in seafood chain. J Food Sci. 84(12):3341–3351. 31769517 10.1111/1750-3841.14915

[CIT0226] Valle J, Lopera E, Sánchez ME, Lerma R, Ruiz JL. 2012. Spontaneous splenic rupture and *Anisakis* appendicitis presenting as abdominal pain: a case report. J Med Case Rep. 6:114. 22524971 10.1186/1752-1947-6-114 PMC3355033

[CIT0227] Vartian C, Septimus E. 1990. Soft-tissue infection caused by *Edwardsiella tarda* and *Aeromonas hydrophila*. J Infect Dis. 161(4):816–816. 2319177 10.1093/infdis/161.4.816

[CIT0228] Vinjé J, Green J, Lewis DC, Gallimore CI, Brown DW, Koopmans MP. 2000. Genetic polymorphism across regions of the three open reading frames of "Norwalk-like viruses". Arch Virol. 145(2):223–241. 10752550 10.1007/s007050050020

[CIT0229] Volpe E, Mandrioli L, Errani F, Serratore P, Zavatta E, Rigillo A, Ciulli S. 2019. Evidence of fish and human pathogens associated with doctor fish (Garrarufa, Heckel, 1843) used for cosmetic treatment. J Fish Dis. 42(12):1637–1638. 31578759 10.1111/jfd.13087

[CIT0230] Weir M, Rajić A, Dutil L, Cernicchiaro N, Uhland FC, Mercier B, Tuševljak N. 2012. Zoonotic bacteria, antimicrobial use and antimicrobial resistance in ornamental fish: a systematic review of the existing research and survey of aquaculture-allied professionals. Epidemiol Infect. 140(2):192–206. 21906415 10.1017/S0950268811001798

[CIT0231] Williams M, Hernandez-Jover M, Shamsi S. 2020. A critical appraisal of global testing protocols for zoonotic parasites in imported seafood applied to seafood safety in Australia. Foods. 9(4):448. 10.3390/foods9040448 PMC7230297 32272621

[CIT0232] Williams M, Hernandez-Jover M, Shamsi S. 2022. Parasites of zoonotic interest in selected edible freshwater fish imported to Australia. Food Waterborne Parasitol. 26:e00138. 34977391 10.1016/j.fawpar.2021.e00138 PMC8686024

[CIT0233] Wimalasena SHMP, Pathirana HNKS, De Silva BCJ, Hossain S, Sugaya E, Nakai T, Heo G-J. 2018. Antibiotic resistance and virulence-associated gene profiles of *Edwardsiella tarda* isolated from cultured fish in Japan. Turk J Fish Aquat Sci. 19(2):141–148.

[CIT0234] Wolfe ND, Dunavan CP, Diamond J. 2007. Origins of major human infectious diseases. Nature. 447(7142):279–283. 17507975 10.1038/nature05775 PMC7095142

[CIT0235] World Health Organization (WHO). 2021. Zoonotic disease: emerging public health threats in the region. http://www.emro.who.int/fr/about-who/rc61/zoonotic-diseases.html.

[CIT0236] Wrobel A, Leo JC, Linke D. 2019. Overcoming fish defense: the virulence factors of *Yersinia ruckeri*. Genes. 10(9):700. 10.3390/genes10090700 PMC6770984 31514317

[CIT0237] Yagoub SO. 2009. Isolation of Enterobacteriaceae and *Pseudomonas* spp. from raw fish sold in fish market in Khartoum state. J Bacteriol Res. 1(7):085–088.

[CIT0238] Yokogawa M, Yoshimura H. 1967. Clinicopathologic studies on larval anisakiasis in Japan. Am J Trop Med Hyg. 16(6):723–728. 6066221

[CIT0239] You HJ, Lee JH, Oh M, Hong SY, Kim D, Noh J, Kim M, Kim BS. 2021. Tackling *Vibrio parahaemolyticus in* ready-to-eat raw fish flesh slices using lytic phage VPT02 isolated from market oyster. Food Res Int. 150(Pt A):110779. 34865794 10.1016/j.foodres.2021.110779

[CIT0240] Yu JE, Cho MY, Kim JW, Kang HY. 2012. Large antibiotic-resistance plasmid of Edwardsiellatarda contributes to virulence in fish. MicrobPathog. 52(5):259–266. 10.1016/j.micpath.2012.01.006 22342431

[CIT0241] Zadoks RN, Barkham T, Crestani C, Nguyen NP, Sirmanapong W, Chen SL. 2020. Population growth, climate change and intensification of the aquaculture industry as drivers of invasive disease emergence in humans in Southeast Asia. The 6th World One Health Congress, 30 October–3 November 2020 Virtual meeting.

[CIT0242] Zahari P, Hirst RG, Shipton WA, Campbell RS. 1990. The origin and pathogenicity of *Basidiobolus* species in Northern Australia. J Med Vet Mycol. 28(6):461–468. 2093119

[CIT0243] Ziarati M, Zorriehzahra MJ, Rezaei H. 2018. *Streptococcus* as an emerging bacterial disease in ornamental fish. In: The second National Conference on Ornamental Fish, 18–19 Feb, Mahalat, Iran. (In Persian)

[CIT0244] Zorriehzahra MEJ, Mehrabi MR, Nazari A. 2014. Can viral nervous necrosis (VNN) disease be considered as a new invasion or new zoonotic disease? Assessing the zoonotic potential of aquatic animal diseases. In: 9th International Symposium on Viruses of Lower Vertebrates, 1–4 Oct 2014, Malaga, Spain.

[CIT0245] Zorriehzahra MJ, Talebi M. 2021. Introduction of bacterial and viral zoonotic diseases of humans and aquatic animals. 4th Congress of Hyrcania Medical Laboratory, Ministry of Health and Medical Education of Iran, Golestan University of Medical Sciences.

